# Bacteriophage Infectivity Against *Pseudomonas aeruginosa* in Saline Conditions

**DOI:** 10.3389/fmicb.2018.00875

**Published:** 2018-05-02

**Authors:** Giantommaso Scarascia, Scott A. Yap, Anna H. Kaksonen, Pei-Ying Hong

**Affiliations:** ^1^Biological and Environmental Science & Engineering Division, Water Desalination and Reuse Center, King Abdullah University of Science and Technology, Thuwal, Saudi Arabia; ^2^Land and Water, Commonwealth Scientific and Industrial Research Organization, Canberra, ACT, Australia

**Keywords:** bacteriophage, green biocides, biofilm removal, planktonic cells, ultrafiltration membrane

## Abstract

*Pseudomonas aeruginosa* is a ubiquitous member of marine biofilm, and reduces thiosulfate to produce toxic hydrogen sulfide gas. In this study, lytic bacteriophages were isolated and applied to inhibit the growth of *P. aeruginosa* in planktonic mode at different temperature, pH, and salinity. Bacteriophages showed optimal infectivity at a multiplicity of infection of 10 in saline conditions, and demonstrated lytic abilities over all tested temperature (25, 30, 37, and 45°C) and pH 6–9. Planktonic *P. aeruginosa* exhibited significantly longer lag phase and lower specific growth rates upon exposure to bacteriophages. Bacteriophages were subsequently applied to *P. aeruginosa*-enriched biofilm and were determined to lower the relative abundance of *Pseudomonas*-related taxa from 0.17 to 5.58% in controls to 0.01–0.61% in treated microbial communities. The relative abundance of *Alphaproteobacteria, Pseudoalteromonas*, and *Planococcaceae* decreased, possibly due to the phage-induced disruption of the biofilm matrix. Lastly, when applied to mitigate biofouling of ultrafiltration membranes, bacteriophages were determined to reduce the transmembrane pressure increase by 18% when utilized alone, and by 49% when used in combination with citric acid. The combined treatment was more effective compared with the citric acid treatment alone, which reported ca. 30% transmembrane pressure reduction. Collectively, the findings demonstrated that bacteriophages can be used as a biocidal agent to mitigate undesirable *P. aeruginosa*-associated problems in seawater applications.

## Introduction

Seawater reverse osmosis (SWRO) desalination has had a great impact on the production of drinking water in the past 40 years (Goosen et al., 2005), and is particularly relied upon by sea-bordered countries that face water scarcity issues (Peñate and García-Rodríguez, 2012). Similar to all membrane-based technologies, SWRO is affected by biofouling. Biofilm formation on membrane detrimentally lowers the flux and salt rejection capacity. In order to maintain the desired desalination performance, plant operators usually apply higher transmembrane pressure which in turn increases energy consumption and economic costs (Fritzmann et al., 2007; Matin et al., 2011).

Bacteria such as *Pseudomonas, Bacillus, Mycobacterium, Acinetobacter* are often detected on fouled RO membranes (Matin et al., 2011). In particular, *Pseudomonas* spp. and *Desulfovibrio* spp. were identified on fouled ultrafiltration (UF) and RO membranes sampled from a SWRO pilot plant located in the Arabian Gulf (Hong et al., 2016). *Pseudomonas* spp. create an optimal niche for *Desulfovibrio* spp. and other sulfate-reducing bacteria (SRB) by depleting oxygen. In addition, SRB and *Pseudomonas aeruginosa* are able to reduce sulfate and thiosulfate, respectively, to produce hydrogen sulfide, a corrosive and toxic gas (Hong et al., 2016). The presence of *Pseudomonas* spp. and SRB in SWRO are favored by the high sulfate concentration prevalent in seawater. In addition, sodium metabisulfite applied to neutralize chlorine within the SWRO (Hong et al., 2016) further provide a source of electron acceptors for these bacterial populations.

To reduce both inorganic and organic foulants on membranes, oxidizing agents such as chlorine, permanganate, and ozone (Fritzmann et al., 2007; Gao et al., 2011) are used in large concentrations. However, the addition of these chemicals in seawater can result in the formation of carcinogenic and toxic disinfection byproducts that can result in public health concerns (Le Roux et al., 2015; Sanawar et al., 2017). Alternatively, regular cleaning of the membrane would have to be performed with acid cleaning (Greenlee et al., 2009). Citric acid cleaning is often used in the pretreatment stage of SWRO to chelate inorganic minerals and disrupt the stability of biofilm matrix attached on the pretreatment UF membranes (Lee et al., 2001; Porcelli and Judd, 2010). Despite their common usage, these biocidal agents have limited penetration through a biofilm matrix and are less effective against biofilm-associated bacteria compared to its effect on planktonic cells (Matin et al., 2011). Moreover, some chemicals e.g., chlorine, can detrimentally impact RO membrane integrity.

To address the concerns arising from conventional biocides and cleaning agents, this study evaluates the use of lytic bacteriophages (i.e., viruses that infect bacteria at high host specificity) to inhibit the growth of planktonic *P. aeruginosa* in saline conditions. In addition, bacteriophages can also induce the release of depolymerases that degrade extracellular polymeric substances (EPS) and hence disrupt biofilm matrix (Harper et al., 2014). It is therefore hypothesized that bacteriophage would be effective to disrupt *P. aeruginosa*-associated biofilms formed on seawater filtration membranes. Although bacteriophage treatment against *P. aeruginosa* biofilm has been performed in therapeutic treatments (McVay et al., 2007; Fu et al., 2010; Alemayehu et al., 2012; Olszak et al., 2015), and as antifouling agents in water and wastewater membrane filtration systems (Zhang et al., 2013; Bhattacharjee et al., 2015), the conditions at which the bacteriophages were applied in those studies differ from those experienced in a SWRO system. Understanding how environmental conditions can impact bacteriophage infectivity is crucial for an effective application of bacteriophages in SWRO systems, where the conditions vary between high pH (i.e., the usual pH of seawater) to low pH (i.e., when citric acid cleaning is performed), high salinity (i.e., in raw seawater) to low salinity (i.e., after seawater is desalinated), and from low (i.e., in the raw seawater) to high temperatures (i.e., seawater retained within the desalination unit).

In this study, the impact of various parameters, namely multiplicity of infection (MOI), pH, salinity and temperature, on the activity of seven lytic bacteriophages against planktonic *P. aeruginosa* cells was analyzed. Three bacteriophages were further applied against a *P. aeruginosa*-enriched seawater biofilm at various pH values and temperatures. Finally, a cocktail of the three bacteriophages was applied alone and in combination with citric acid cleaning to cross-flow seawater ultrafiltration setup, and the membrane modules were assessed for their transmembrane pressure and biofilm cell counts.

## Materials and methods

### Bacteriophage isolation

Bacteriophages were isolated from an influent collected from a wastewater treatment plant located in KAUST. A 50 mL aliquot of the influent was centrifuged at 8,500 *g* for 20 min and thereafter supernatant was filtered through 0.22 μm cellulose acetate syringe filter (VWR, Radnor, PA) to remove bacterial cells. The filtrate was mixed with 50 mL Lennox broth (LB) supplemented with 35 g/L NaCl and 4 mM Ca^2+^, and 50 mL of exponentially growing *P. aeruginosa* strain DSM1117. The mixed culture was incubated for 24 h at 37°C. Thereafter, 1% v/v chloroform was added to lyse the bacterial cells, and incubated for 2 h at room temperature with constant agitation at 100 rpm. The culture was centrifuged at 8,500 *g* for 30 min at 4°C and the supernatant was filtered through 0.22 μm syringe filter to remove bacterial cells. Several dilutions, ranging from 10^−3^ to 10^−5^ fold, were performed in sodium magnesium (SM) buffer (5.8 g/L NaCl, 0.975 g/L MgSO_4_, 50 ml/L 50 mM Tris-Cl 7.5 pH) and 10 μL of each diluted sample was mixed with 100 μL of *P. aeruginosa* culture to detect the presence of plaques using the double layer plate method (Adams, 1959). Seven plaques were isolated with sterile inoculating loops, transferred to SM buffer and filtered through 0.22 μm syringe filter to remove bacterial cells. The bacteriophages from the seven plaques were named P1 through P7 and propagated 10 more times using the above mentioned procedure.

### Infection of planktonic cultures with bacteriophages at various conditions

All bacteriophages were tested for their infectivity against other bacterial species using the soft agar plaque assay method. All the bacteria used in this study are listed in Table 1. After verifying their host specificity, bacteriophage activity against *P. aeruginosa* was analyzed at various multiplicity of infection (MOI) values, namely 0.1, 1, and 10. MOI represents the ratio between the number of bacteriophage particles determine as phage forming units (PFU/mL) and bacterial cells determined as colony forming units (CFU/mL) in a mixture of 1:1 LB broth and SM buffer, pH 7. MOI was calculated for each bacteriophage based on the number of plaques formed on agar (Figure S1). For each MOI, one control was prepared without bacteriophages. The treated cultures and controls were incubated at 37°C with constant shaking at 200 rpm for 24 h. Growth curves of *P. aeruginosa* were obtained with and without the presence of the seven phages. The best MOI value in terms of bacterial inhibition time (in h) and specific growth rate reduction (h^−1^) compared with the control was selected for evaluating the impact of other parameters. Subsequently, bacteriophage infectivity against *P. aeruginosa* cultures was evaluated at different temperatures, pH, and salinity, with the same procedure described above at a constant MOI of 10. All experiments were performed in triplicates.

**Table 1:** Name and origin of the bacteria used in this study.

**Bacteria**	**Origin**
*Pseudomonas aeruginosa* DSM1117	Blood culture
*Pseudomonas stutzeri* CE9	Chlorinated wastewater effluent (Jeddah, Saudi Arabia; Al-Jassim et al., 2015)
*Pseudomonas otitidis* CP2	
*Pseudomonas resinovorans* CE5	
*Pseudomonas pseudoalcaligenes* CE3	
*Aeromonas hydrophila* subsp. *anaerogenes* CP3	
*Aeromonas veronii* CP10	

### Infection of biofilm with bacteriophages at various conditions

To examine the effect of bacteriophages on the biofilm structure at various temperatures (25, 30, 37, and 45°C) and pH values (5, 6, 7, 8), 28 drip flow reactors (BioSurface Technologies Corp., Bozeman, MT) were assembled. Twenty eight cellulose acetate ultrafiltration membranes (Sterilitech Corporation, Kent, WA), each with surface area of 11.25 cm^2^, were secured on glass coupons and placed into the drip flow reactors to establish biofilm on the membranes. Reactors were covered with aluminum foil to avoid light exposure. Seawater from Red Sea was fed into each reactor at a continuous rate of 5 mL per minute. The feed was replaced with fresh seawater every 3 d. After 4 weeks, the feed solution was replaced with 5 L of *P. aeruginosa*-enriched seawater. Briefly, *P. aeruginosa* was cultivated overnight on *Pseudomonas* isolation agar (Sigma-Aldrich, St Louis, MO); single colonies were transferred into multiple tubes with 20 mL LB broth and grown at 37°C with constant shaking (200 rpm) to reach OD_600_ of 0.7. After centrifugation at 4,100 *g* for 30 min, bacterial pellet was resuspended in 5 L seawater to achieve a cell density of 3.5 × 10^7^ CFU/mL of *P. aeruginosa*. This feed was also replaced every 3 d.

After 4 weeks, to calculate the starting number of *Pseudomonas* colonies, 4 membranes were removed from the reactor and washed twice in 1× phosphate-buffered saline (PBS) to remove loosely attached biofilm. Membranes were cut into 16 identical pieces and were placed individually into collection tubes containing 2 mL 1× PBS. Attached biofilm was sonicated for 5 min by a Q500 sonicator (Qsonica, Newton, CT, US) at 30% amplitude with 3 s pulsating steps to detach biomass from the membranes. The supernatant was serially diluted for spread plating on *Pseudomonas* isolation agar and incubated at 37°C for 1 d for colony counting. The remaining membranes were removed, rinsed twice in 1× PBS to remove loosely attached biofilm, and aseptically cut into four pieces for a total of 96 identical fragments. Each piece was transferred to an individual 15 mL tube containing LB broth and SM buffer (each in 50% v/v, salinity 3.5%). Bacteriophages P1, P5, and P7 were propagated as described above and were diluted with SM buffer to the adequate number of PFU/mL to attain an MOI value of 10. Each bacteriophage was then used individually to infect attached biofilm on the membrane at four different temperatures (25, 30, 37, and 45°C) and pH values (5, 6, 7, 8) for 10 h at constant shaking of 200 rpm. A control without bacteriophage inoculation was prepared for each temperature and pH condition. After 10 h of phage infection, biofilm was sonicated in 3.5 mL of 1× PBS and analyzed based on procedures as described in sections *P. aeruginosa* Colony and Bacteriophage Plaque Counts on Membranes and RNA Extraction and 16S rRNA Gene Based High-Throughput Sequencing. All experiments were conducted in triplicate.

### *Pseudomonas aeruginosa* colony and bacteriophage plaque counts on membranes

A 100 μL aliquot of bacterial suspension was used to evaluate the number of colony forming units (CFU)/cm^2^ on *Pseudomonas* isolation agar, while 1 mL was filtered through 0.22 μm syringe filter to assess the number of plaque forming units (PFU)/cm^2^ recovered from the different tested conditions.

### RNA extraction and 16S rRNA gene based high-throughput sequencing

A 2 mL aliquot of the biofilm suspension (as described in section Infection of Biofilm With Bacteriophages at Various Conditions) was utilized for RNA extraction. To avoid RNA degradation, 4 mL of RNAprotect® Cell Reagent (Qiagen, Hilden, Germany) was added to each bacterial suspension immediately after sampling. The mixture was incubated at room temperature for 5 min and then centrifuged for 10 min at 5,400 *g*. After centrifugation, the supernatant was removed and the pellet was stored at −80°C for ~2 weeks until RNA extraction.

RNA extraction from the biomass pellet was performed using the RNeasy® Midi Kit (Qiagen, Hilden, Germany) following manufacturer's protocol and RNA concentration was measured with the Invitrogen RNA HS Qubit® 2.0 assay kit (Thermo Fisher Scientific, Carlsbad, CA). Extracted RNA was reverse transcribed into first-strand complementary DNA (cDNA) using the Invitrogen SuperScript^TM^ First-Strand Synthesis System (Thermo Fisher Scientific, Carlsbad, CA). The cDNA was then used as template to amplify for 16S rRNA genes with primer pair 515F (5′- Illumina overhang- GTG YCA GCM GCC GCG GTA A-3′) and 907R (5′- Illumina overhang- CCC CGY CAA TTC MTT TRA GT-3′) based on the procedure described earlier (Scarascia et al., 2017). Purified amplicons were submitted to KAUST Genomic Core lab for amplicon sequencing on Illumina MiSeq platform. All high-throughput sequencing files used in this study are deposited in the European Nucleotide Archive (ENA) and are accessible via accession number PRJEB23782.

### Biofilm microbial community data analysis

Amplicon sequences were sorted on a Phred score >30 and primers, adaptors, and index sequences were removed. After removing any sequences longer than 280 nt, sequence chimeras were identified and removed by UCHIME (Edgar et al., 2011). Chimera-free sequences were split and a subset containing 100,000 sequences were further analyzed for each sample. Taxonomical assignment was obtained at 95% classification reliability level with copy number adjustment using the Ribosomal Database Project (RDP) Classifier (Wang et al., 2007). Relative abundance at genus and phylum level was calculated for each sample. Special emphasis was made to determine the relative abundance of *Pseudomonas*-related taxa in the presence of bacteriophages compared to the control. Chimera-removed sequence files were also sorted for unique operational taxonomic units (OTUs) at 97% 16S rRNA gene similarity [27]. Similarity percentages (SIMPER) analysis was performed using Primer-E version 7 to identify OTUs that showed a significant difference in their relative abundance between infected and non-infected biofilm (Clarke, 2015). The OTU identities were checked against the NCBI nucleotide database using the BLASTN algorithm.

### Effect of bacteriophage treatment on UF membrane fouling

A UF filtration system was set up as illustrated in Figure S2. Five UF cellulose acetate membranes of pore size 8 kDa (Sterilitech Corporation, Kent, WA) were aseptically cut into dimensions of 10 by 2.5 cm each, soaked in deionized water for 24 h, and then rinsed in 80% ethanol. Sterile membranes were then individually placed inside cassette modules. Subsequently, deionized water was circulated through the modules to reach a stable trans-membrane pressure (TMP_0_). The modules were operated in cross-flow mode at room temperature with the concentrate recirculating into the feed tank. A constant flux of 12 L/m^2^/h (LMH) was maintained. Artificial seawater (26.29 g/L NaCl, 0.74 g/L KCl, 1.32 g/L CaCl_2_ dehydrated, 6.09 g/L MgCl_2_·7H_2_O, 1.92 g/L MgSO_4_, pH 7.8) was used as feed solution. Nutrient broth (HiMedia, Mumbai, India) was added to the feed solution to obtain a nutrient concentration of 24 mg/L as described previously (Oh et al., 2017). Feed bottle was inoculated with *P. aeruginosa* culture at a final concentration of 5 × 10^7^ CFU/mL and this feed was replaced every 3 d. Biofilm was established on each membrane for 6 d with a recording of the transmembrane pressure (TMP) every 8 h.

On the 7th day, the filtration system was kept in offline mode for four different cleaning treatments: (i) 5 × 10^8^ PFU/mL phage cocktail application in SM buffer for 6 h followed by deionized water for 3 h, (ii) 0.1 M citric acid treatment alone for 3 h followed by deionized water for 6 h, (iii) 5 × 10^8^ PFU/mL phage cocktail application in SM buffer for 6 h followed by 0.1 M citric acid treatment for 3 h, and (iv) 0.1 M citric acid treatment for 3 h followed by 5 × 10^8^ PFU/mL phage cocktail application in SM buffer for 6 h. One membrane was used as non-treated control where deionized water was applied for the same 9 h duration as that of the treatments. All membranes were flushed for 10 min with deionized water at the end of the treatments, and the TMP monitoring was resumed after 1 h from the treatment. Cleaning cycles were repeated every 3 d for a total of three cycles.

At the end of the third cycle, membranes were removed from the modules and three 2 × 2 cm pieces were cut from each membrane sheet and placed in 2 mL 1× PBS to enumerate for the *Pseudomonas* colony counts and total cell counts. Cells were stained with SYBR® green (Thermo Fisher Scientific, Waltham, MA, US) and counted by BD Accuri C6 flow cytometer (BD, Bioscience, NJ, US). The cleaning experiment on the filtration membranes was performed in duplicate as run 1 and run 2.

### Bacteriophages morphology and genome size characterization

Pure bacteriophages P1, P5, and P7 in cultures were fixed with 2.5% v/v glutaraldehyde. A 5 μL aliquot of each bacteriophage culture was deposited on carbon-coated copper grids and negatively stained with 1% w/v uranyl acetate (pH 4.5). Samples were washed with sterile water, air-dried, and visualized through transmission electron microscopy (TEM) (Tecnai Spirit TWIN, FEI) operated at 120 kV and equipped with an ORIUS SC1000 camera (Gaitan). Image analysis was carried out using ImageJ Software.

Direct phage plaques DNA was extracted for the three bacteriophages as described previously (Kot et al., 2014). Briefly, plaques were resuspended in DNase I buffer, filtered and treated for 30 min with DNase I; DNase was inactivated by 10 μL of 50 mM EDTA and further treated with Proteinase K (Thermo Scientific, Waltham, USA) before DNA extraction using UltraClean Soil DNA Isolation Kit (MoBio Laboratories, Carlsbad, USA) based on manufacturer's protocol. Bacterial DNA contamination was checked through 16S rRNA gene amplification and all plaque DNA samples showed no amplification for 16S rRNA genes. Extracted DNA was used to assess genome size through pulsed field gel electrophoresis (PFGE). DNA samples were run on 1% w/v agarose gel using the CHEF-DR III system (Bio-Rad, Hercules, CA, USA) (18 h, 6 V cm^−1^, 14°C, angle 120°, switch time 0.1–1). *Lambda* DNA Ladder (New England, Biolabs, Ipswich, MA, USA) was used as size marker.

### Statistical analysis

One-way ANOVA was performed to evaluate for statistical differences between treatments. Statistical differences were considered significant at 95% confidence level (*p* < 0.05).

## Results

### Bacteriophage infectivity against planktonic *Pseudomonas aeruginosa*

**Figure 1:**
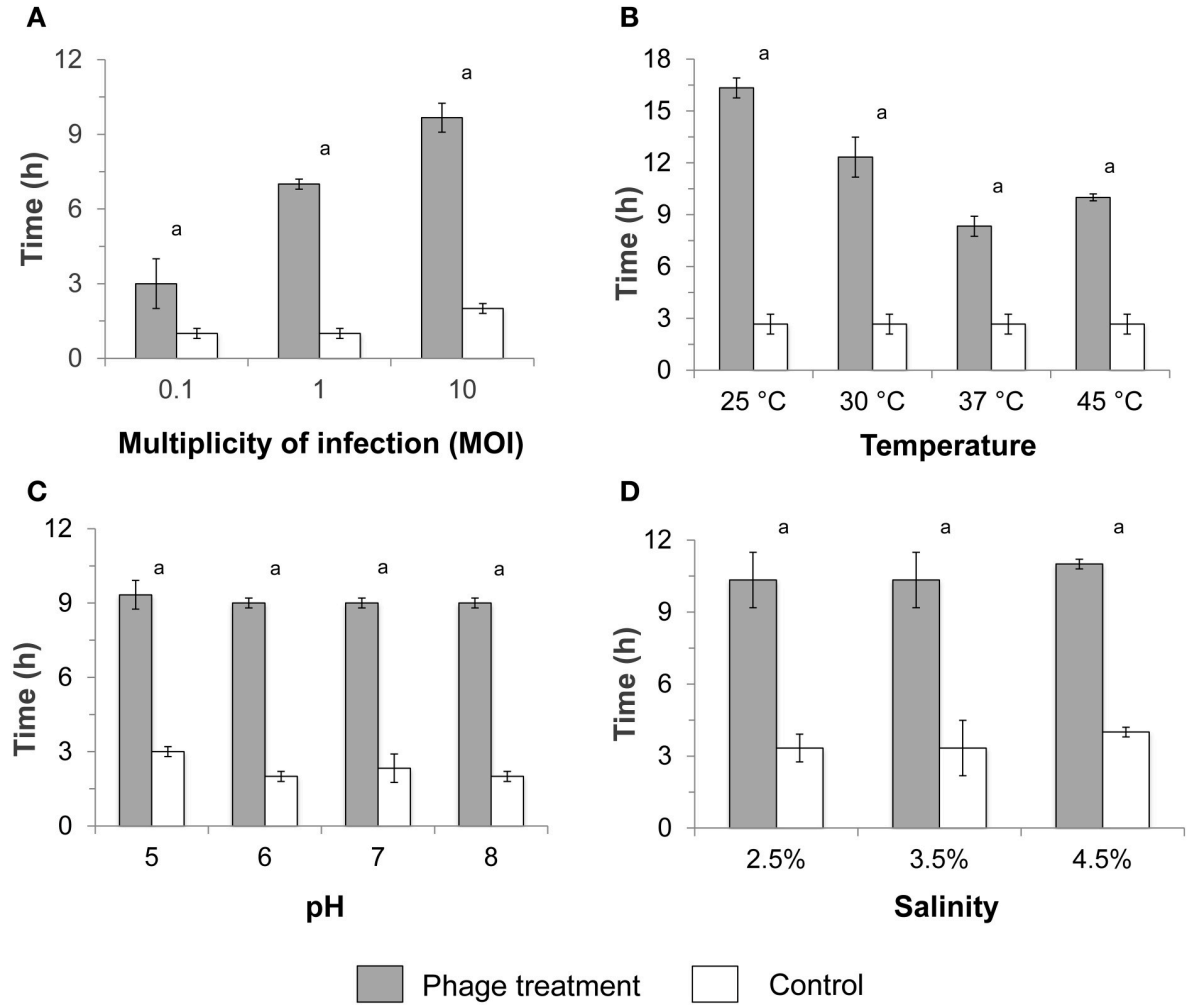
Average duration of the lag phase in infected and non-infected (control) planktonic *Pseudomonas aeruginosa* culture after phage treatment at various **(A)** multiplicities of infection (MOIs), **(B)** temperatures, **(C)** pH values, and **(D)** salinities. The standard test conditions were MOI 10, 37°C, pH 7, and salinity 3.5% when these parameters were not varied. Bars indicate the standard deviation among the three biological replicates. Letter a indicates statistical difference between each treatment and the respective control at *p* < 0.0006.

Plaques were only observed when isolated bacteriophages were infected against *P. aeruginosa* but not against all other tested bacterial hosts (Table 1). Infectivity of the seven isolated bacteriophages against *P. aeruginosa* growing in planktonic phase was systematically analyzed at various MOIs, temperatures, pH values, and salinities based on lag phase duration and specific growth rates. Lag phase of non-infected control cultures ranged from 1 to 4 h (Figures 1A–D), while specific growth rates ranged from 0.21 to 0.40 h^−1^ (Table 2). In contrast, the lag phase duration of the infected bacterial culture was significantly longer compared to the control (*p* < 0.0001). The longer lag phase duration was observed regardless of the bacteriophage applied.

**Table 2:** Average specific bacterial growth rate (h^−1^) for infected *Pseudomonas aeruginosa* culture in the presence of bacteriophages (P1–P7) and in non-infected culture (C).

**Variable**	**Value**	**P1**	**P2**	**P3**	**P4**	**P5**	**P6**	**P7**	**C**
MOI^a^	0.1	0.23	0.22	0.22	0.23	0.23	0.22	0.24	0.21
	1	0.28	0.24	0.27	0.23	0.25	0.24	0.25	0.29
	10	0.22	0.21	0.21	0.22	0.21	0.21	0.22	0.34
Temperature (°C)^b^	25	0.25	0.21	0.22	0.22	0.23	0.23	0.23	0.34
	30	0.30	0.28	0.28	0.26	0.27	0.26	0.28	0.38
	37	0.21	0.22	0.20	0.21	0.20	0.19	0.21	0.34
	45	0.20	0.19	0.19	0.20	0.22	0.21	0.22	0.29
pH^c^	5	0.20	0.21	0.18	0.19	0.22	0.23	0.22	0.34
	6	0.17	0.19	0.18	0.20	0.19	0.19	0.18	0.34
	7	0.21	0.26	0.22	0.22	0.23	0.26	0.20	0.33
	8	0.28	0.32	0.27	0.31	0.26	0.29	0.30	0.40
Salinity (%)^d^	2.5	0.25	0.23	0.24	0.23	0.24	0.24	0.24	0.38
	3.5	0.23	0.22	0.22	0.21	0.22	0.20	0.22	0.36
	4.5	0.24	0.24	0.22	0.20	0.21	0.20	0.24	0.32

*Specific growth rates were determined at various MOIs (0.1, 1 and 10), temperatures (25, 30, 37, and 45 °C), pH values (5, 6, 7, and 8) and salinities (2.5, 3.5, and 4.5%). Gray shades indicate statistically lower growth rates (p < 0.05 green, p < 0.007 blue, and p < 0.0006 red) compared with the related control*.

a Temperature 37°C, pH 7, salinity 3.5%;

b MOI 10, pH 7, salinity 3.5%;

c MOI 10, temperature 37°C, salinity 3.5%;

d *MOI 10, temperature 37°C, pH 7*.

At a MOI of 10, the difference in the lag phase duration between bacteriophage-treated culture and control was the highest (7.7 h; Figure 1A). Bacterial cultures infected with a MOI of 10 also showed significantly lower specific growth rates (*p* < 0.0003) compared with the control (Table 2). Infection at MOI 0.1 did not result in any specific growth rate reduction, while a MOI of 1 for phage P4 and P6 was able to reduce specific growth rates (Table 2). As such, subsequent experiments to test infectivity at various temperatures, pH values and salinities were conducted at a MOI value of 10.

Lag phase was at least 3 times longer in bacteriophage-treated cultures compared with the control over all temperatures tested (*p* < 0.0001) (MOI 10, pH 7, salinity 3.5%), with particularly longer lag phase at 25 and 30°C (Figure 1B). The lag phase was followed by a bacterial growth with lower specific rates compared with the control. Significant reductions in specific growth rates were observed at 25, 30, 37°C (*p* < 0.0003) and 45°C (*p* < 0.03) compared with the respective controls.

A longer lag phase of ca. 9 h was observed in the presence of bacteriophage regardless of the pH at a constant MOI of 10, 37°C, and 3.5% salinity (*p* < 0.0001; Figure 1C), compared to the ca. 3 h lag phase for controls. Specific growth rate reduction was also observed when *P. aeruginosa* was infected at various pH values, with a higher decrease at pH 5 and 6 (*p* < 0.0005) compared with infection at pH 7 and 8 (*p* < 0.045; Table 2). Similarly, the lag phase duration was significantly prolonged to ca. 10 h (*p* < 0.0001) at various salinities in the presence of bacteriophages (Figure 1D). One-way ANOVA test showed significant reduction in specific growth rates in the presence of bacteriophages compared to controls at all tested salinities (*p* < 0.007).

### Bacteriophage infectivity against *P. aeruginosa*-enriched biofilm

**Figure 2:**
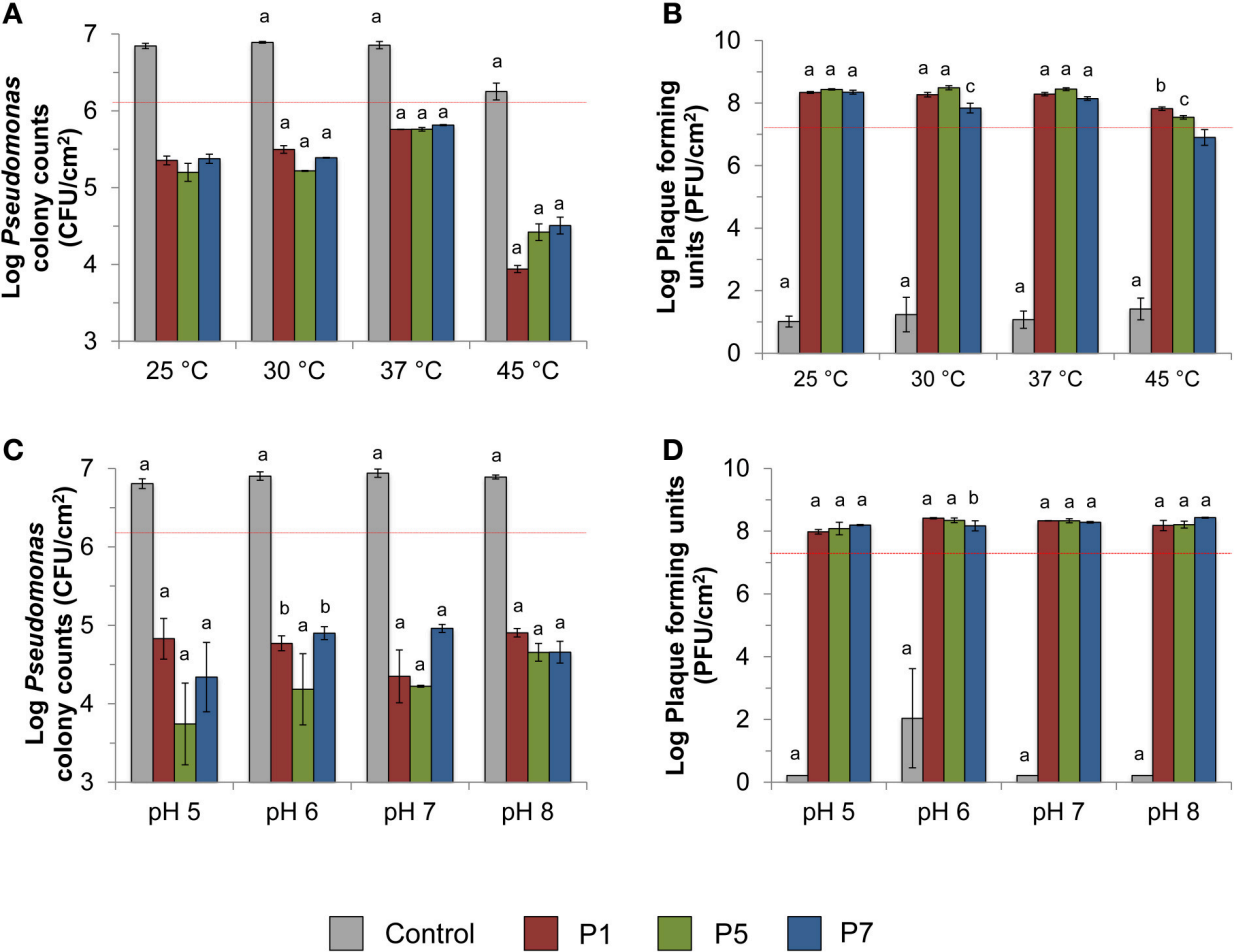
Effect of phage treatment against *Pseudomonas aeruginosa* enriched biofilm. The effect was evaluated in terms of **(A)** viable colony counts on *Pseudomonas* isolation agar at various temperatures, **(B)** recovery of plaque counts after 10 h bacteriophage exposure at various temperatures, **(C)** viable colony counts on *Pseudomonas* isolation agar at various pH values, and **(D)** the recovery of plaque counts after 10 h bacteriophage exposure at various pH values. Dashed red lines indicate the number of CFU before infection or the number of PFU spiked for biofilm infection. The standard test conditions were MOI 10, 37°C, pH 7, and salinity 3.5% when these parameters were not varied. Bars indicate standard deviation among the three biological replicates. Letters indicate statistical difference between each treatment and the original spiked amount as indicated by red dash lines (a: *p* < 0.0006, b: *p* < 0.007, c: *p* < 0.05).

*Pseudomonas aeruginosa*-enriched seawater biofilm was infected separately with three bacteriophages (P1, P5, and P7) at four different temperatures (25, 30, 37, and 45°C) and pH values (5, 6, 7, and 8) for 10 h. At all tested temperatures and upon infection with bacteriophages, the number of viable bacterial cells recovered from *Pseudomonas* isolation agar decreased significantly to an average of 2.8 × 10^5^ CFU/cm^2^ (*p* < 0.0001) compared with the initial number (1.37 × 10^6^ CFU/cm^2^) recovered from the membranes (Figure 2A). In contrast, the controls increased significantly to an average of 6.0 × 10^6^ CFU/cm^2^ and the number of bacterial colonies was significantly higher than the initial number (*p* < 0.04). Most of the bacteriophages, specifically P7, showed lower PFU recovery at high temperature of 45°C compared with the other temperatures (Figure 2B). Bacteriophage P7, in particular, had significantly lower PFU counts than the other bacteriophages at 45°C. The number of PFU counts for P7 was also significantly different from the PFU counts obtained after infection at 25, 30, and 37°C (*p* < 0.0001 Figure 2B).

At all tested pH values, the number of bacterial colonies in infected biofilm decreased significantly to an average of 4.9 × 10^4^ CFU/cm^2^ compared with the initial spiked numbers of 1.7 × 10^6^ CFU/cm^2^ (*p* < 0.001; 1.5–2 logs reduction). This is in contrast with the bacterial cell count observed for the controls, where the number of bacterial colonies was significantly higher than the initial numbers (*p* < 0.0001; Figure 2C). Phages were able to replicate by 1.6–13 times of the initial number (1.7 × 10^7^ PFU/cm^2^) at all tested pH values (*p* < 0.05; Figure 2D).

### The effect of phage treatment on biofilm microbial community based on 16S rRNA gene-based amplicon sequencing data analysis

Overall *Proteobacteria* was the most abundant phylum with an average relative abundance of 95%. At genus level, *Vibrio* and unclassified *Vibrionaceae* together accounted for ca. 34.5–65% of the microbial community regardless of the tested condition. *Pseudoalteromonas* was found to be present at high relative abundance of up to 37.3% of total microbial community, while *Alteromonas, Arcobacter*, and *Thalassospira* showed a relative abundance lower than 10%. Unclassified *Pseudomonadaceae* and *Pseudomonas* spp. were present in all samples at an average relative abundance of 0.75 and 0.62%, respectively. The relative abundance of these unclassified *Pseudomonadaceae* and *Pseudomonas* spp. in infected biofilm ranged from 0.01 to 0.61% of the total community, while in the controls they ranged from 0.17 to 5.58% (Table 3). Specifically, at 25, 30, and 45°C, the relative abundance of both *Pseudomona*s-related taxa in bacteriophage-treated samples was significantly lower compared with the control (*p* < 0.05). Similarly, when the same experiment was carried out at various pH values, the average of relative abundance of both taxa was significantly lower in bacteriophage-infected samples compared with the controls at all tested pH values (*p* < 0.03).

**Table 3:** Average of the relative abundance in percentage among the three biological replicates of two *Pseudomonas*-related taxa (Unclassified *Pseudomonadaceae* and *Pseudomonas*).

**Variable**	**Value**	**Unclassified** ***Pseudomonadaceae***				***Pseudomonas***			
		**P1**	**P5**	**P7**	**C**	**P1**	**P5**	**P7**	**C**
Temperature (°C)^a^	25°C	0.05	0.06	0.07	0.27	0.05	0.07	0.08	0.27
	30°C	0.01	0.04	0.01	0.29	0.02	0.07	0.02	0.27
	37°C	0.12	0.14	0.12	0.17	0.18	0.18	0.15	0.21
	45°C	0.61	0.43	0.32	2.50	0.59	0.38	0.29	2.78
pH^b^	5	0.13	0.07	0.13	5.58	0.10	0.06	0.11	4.26
	6	0.20	0.18	0.30	3.72	0.17	0.14	0.26	2.48
	7	0.1	0.08	0.23	3.23	0.07	0.08	0.18	2.46
	8	0.15	0.13	0.24	4.40	0.13	0.08	0.21	3.50

*Gray shade indicates statistically higher average relative abundance of the related taxa in the control (C) compared with biofilm infected with bacteriophages P1, P5, or P7*.

a MOI 10, pH 7, salinity 3.5%;

b *MOI 10, temperature 37°C, salinity 3.5%*.

From SIMPER analysis, the average dissimilarity between infected biofilm and control ranged from 13.8 to 17.4%. Unclassified *Pseudomonadaceae* and *Pseudomonas* accounted for the three main populations impacted by bacteriophages (Table S1). Other genera affected by bacteriophages were *Pseudoalteromonas*, unclassified *Alphaproteobacteria*, unclassified *Planococcaceae*, and unclassified *Rhodobacteraceae* (Table S1). An OTU-based analysis of biofilm infected at various temperatures and pH values further showed that the only OTU associated with genus *Pseudomonas* that was significantly impacted by the phage treatment shared at least 98% similarity with *P. aeruginosa* (Table S2). Finally, an OTU of at least 98% similarity with *Pseudoalteromonas shioyasakiensis* along with other OTUs related to *Thalassospira, Alcanivorax*, and *Aestuatiibacter* genera were also affected by bacteriophage application (Table S2).

### Effects of ultrafiltration (UF) membrane cleaning treatments on transmembrane pressure (TMP)

**Figure 3:**
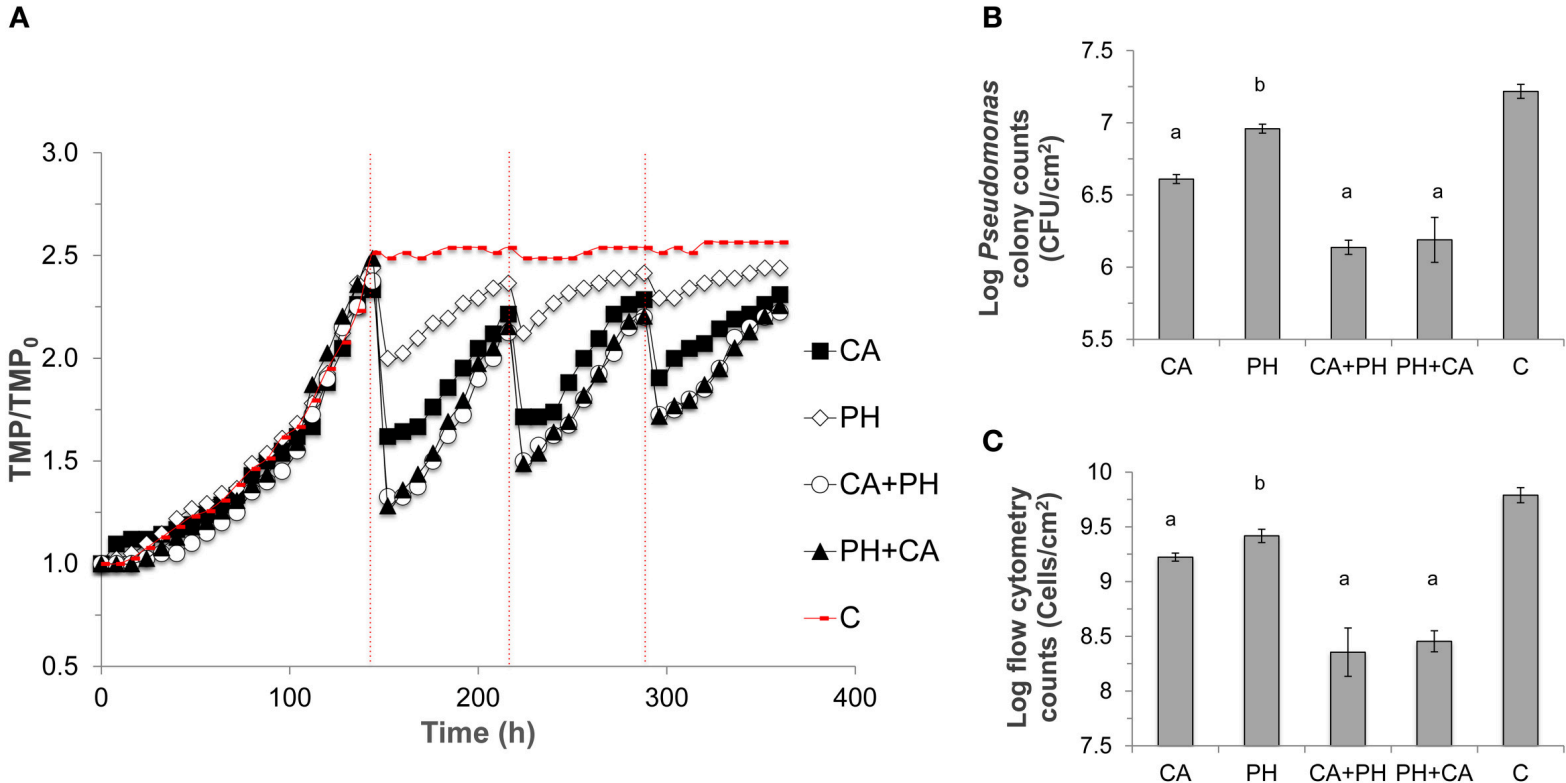
Run 1 of the ultrafiltration membrane experiment. **(A)** Transmembrane pressure (TMP) profiles of ultrafiltration membranes. TMP was normalized against the initial TMP (TMP_0_) for each membrane. Different treatments were applied, namely CA, Citric acid; PH, Phage; CA+PH, Citric acid followed by phage; PH+CA, Phage followed by citric acid; C, no treatment. Dashed red lines indicate the point at which treatment was applied over three different cycles. **(B)** Bacterial sell numbers from plate counts in terms of CFU/mL, **(C)** and from flow cytometry in terms of cell number/mL, were measured on three 2 × 2 cm pieces for each membrane at the end of the experiment. Bars indicate standard deviation among the three biological replicates. Letters indicate statistical difference compared with the control (a: *p* < 0.0006, b: *p* < 0.007).

Non-treated UF membrane showed a rapid increase in its TMP, and reached critical fouling after 130 h of operation. When the first cleaning cycle was implemented at 144 h, the membrane treated with citric acid alone showed a 30% reduction in TMP, while bacteriophage treatment alone resulted in 18% reduction. Bacteriophage in combination with citric acid, regardless of the sequence of treatment, resulted in a TMP decrease of ca. 49%. The same trend was observed for all subsequent cleaning cycles, albeit with decreasing efficacy toward TMP drop with every cycle (Figure 3A). The slopes of TMP increase exhibited by membranes subjected to bacteriophage cleaning alone for all cleaning cycles were significantly lower than those of other treatments (Paired *t*-test, *P* < 0.01, Figure S3). There was no statistical difference between the slopes of TMP increase for the respective treatment among the three cleaning cycles (Figure S4).

The lower TMP was due to a lower average number of bacterial cells attached on the membranes after the cleaning treatment. To exemplify, the average number of bacterial cells on non-treated membrane fragment was 8.3 × 10^6^ CFU/cm^2^. Instead, all treatments significantly reduced both the bacterial colony counts and total cell counts (*p* < 0.0001), with the highest log reduction of 1.1-logs observed when the combined cleaning was conducted (Figures 3B,C). Lower TMP and cell counts were also observed in the duplicate experiment (Figure S5).

### Bacteriophage genome size and morphology

The genome sizes of bacteriophages P1, P5, and P7 were determined through pulse field gel electrophoresis (Figure S6), and were observed to share a similar genome size of approximately 48 kbp. This genome size fits in the reported range of both *Podoviridae* and *Siphoviridae* families (41.6–79.4 and 34.5–61.1 kbp, respectively; Pires et al., 2015).

Bacteriophages P1, P5, and P7 were further characterized for their morphology through transmission electron microscopy (TEM). Bacteriophages P1 and P5 had similar morphology, both had an icosahedral head of ~55 and ~47 nm, respectively, and short tail of ~12 and ~9 nm, respectively (Figures S7, S8). Phage P7 was often found in agglomerates (Figure S9) and showed ~60 nm icosahedral head and with no easily identifiable tail (Figure S10). Following the Ackermann classification (Ackermann, 2006), these morphological traits suggest that the three bacteriophages could possibly belong to the order *Caudovirales*, family *Podoviridae*, as characterized by icosahedral head with short or no tail.

## Discussion

In this study, the feasibility of applying bacteriophages to mitigate biofouling caused by *P. aeruginosa* in applications involving seawater is investigated. Infectivity against *P. aeruginosa* in both planktonic phase and biofilm were systematically studied under a range of salinity, temperature, and pH conditions that are representative of different stages of seawater desalination process. In addition, bacteriophages when applied in combination with conventional citric acid cleaning procedure were found to be effective in reducing the transmembrane pressure increment.

To infect bacterial hosts, bacteriophages first attach to host cells and integrate their DNA into the host genome without killing the hosts. Subsequently, in response to environmental or other signal cues from the bacterial hosts (Echols, 1972), bacteriophages then enter into lytic infection mode and result in the death of bacterial hosts through the induction of a suite of proteins including holins, endolysins, and spanins. These proteins collectively disrupt the cell membranes and achieve cell lysis (Young, 2013, 2014). Furthermore, bacteriophages possess enzymes that degrade the extracellular polymeric substances of biofilm matrix (Harper et al., 2014), in turn dispersing the biofilm structure to increase the susceptibility of bacterial cells to biocides or chemical cleaning. This is especially relevant for tackling membrane fouling issues in SWRO plants since traditional chemical cleaning and chlorination have limited effect in eradicating bacteria such as *Pseudomonas* spp. and SRB (Khan et al., 2015; Hong et al., 2016), both of which have been found to be associated with membrane fouling and production of corrosive hydrogen sulfide (Hong et al., 2016).

Most host-specific bacteriophages like the ones isolated in this study exhibit lytic actions that are specific against a limited number of hosts, and the use of host-specific bacteriophages can potentially minimize unintentional ecological impacts on the indigenous microbial community present within the ecosystem. However, to facilitate the use of bacteriophages as antifouling agent for seawater applications, parameters such as bacteriophage-host ratio, temperature, pH, and salinity need to be taken in consideration. In natural marine environments, bacteriophages distribution and proliferation are determined by the productivity and density of the specific host population. In our study, the highest lag phase extension and slowest specific growth rate were also observed for *P. aeruginosa* infected at a MOI of 10 (Figure 1A and Table 2) and in saline media (Figure 1D and Table 2). In natural marine environments, bacteriophages distribution and proliferation are determined by the productivity and density of the specific host population. In this study, a MOI value of 10 was the optimal for bacteriophage infection. It is likely that this MOI value maximized encounter rates between bacteriophage and bacterial cell, hence ensuring that nearly all host cells are infected by at least one bacteriophage particle.

Besides an optimal bacteriophage-host ratio, temperature plays a fundamental role in governing the attachment, proliferation, and cell lysis efficiencies. Attachment is dependent on capsid proteins of bacteriophages and surface proteins on bacterial hosts, both of which are sensitive to high temperatures. The findings of this work suggest that the isolated bacteriophages were able to infect within a broad temperature range of 25–45°C. However, the highest tested temperature resulted in a lower reduction in specific growth rates of *P. aeruginosa* compared to the other temperatures (Table 2). The lower lytic efficiency at 45°C can be accounted for by a lower bacterial growth and replication (Figure 2A), which may have provided lower surface area for bacteriophage adsorption (Sillankorva et al., 2004). Also, the high temperature of 45°C affected bacteriophage replication as evidenced from the lower PFU recovery compared to other temperatures (Figure 2B). Among the isolated bacteriophages, P7 seemed to be particularly sensitive to high temperature of 45°C. The PFU recovery for this bacteriophage showed a complex trend in which it first decreased at 30°C compared with the one at 25°C (*p* = 0.004) and it subsequently increased again at 37°C (*p* = 0.04) before decreasing at 45°C (*p* < 0.0001). The exact reasons to account for this cyclical behavior are not known. However, it may be possible that this bacteriophage was more affected than P1 and P5 by the different host metabolic activity at different temperatures. Bacterial density and metabolic activities are, in fact, key factors for an efficient bacteriophage proliferation (Chibani-Chennoufi et al., 2004). High temperatures can also result in lower infection efficiency due to alteration made in the capsid proteins and bacterial surface receptors, hence inhibiting the initial phage attachment (Sillankorva et al., 2004). Similarly, Kwiatek and colleagues observed a 1 order of magnitude reduction in the virus particles concentration when bacteriophages from *Podoviridae* family were maintained at 50°C (Kwiatek et al., 2015).

In addition to temperature, pH is another important parameter that affects bacteriophage activity. Environmental pH can change the charge state of the amino acids in bacteriophage capsids, which in turn lead to modifications in the capsid structure (Nap et al., 2014). Moreover, at pH close to the isoelectric point, virus aggregation was observed in MS2 bacteriophage (Langlet et al., 2007), which can contribute to significant bias during PFU enumeration. Despite this, the pH range analyzed in this study exhibited no apparent effect on bacteriophage infectivity against both planktonic cells (Figure 1C, Table 2) and biofilm (Figures 2C,D), suggesting the applied pH was feasible for application in SWRO, which is generally operated within this pH range.

In an earlier study, bacteriophages were stored at a similar pH range and were determined to be stable (Kwiatek et al., 2015). Coincidentally, both in this and the earlier study, isolated lytic bacteriophages were possibly associated with the *Podoviridae* family. *Podoviridae* were shown to survive large temperature fluctuations compared to bacteriophages with smaller genome sizes (Jonczyk et al., 2011). A complete genome sequencing of the isolated bacteriophages is not done for this study and it was therefore not possible to provide better and definitive information about bacteriophage classification and infection mechanisms at different conditions. However, based on morphological traits, *Podoviridae* are generally characterized by a big icosahedral head and short tail, which were observed for the bacteriophages in this study. These phenotypic characteristics observed may have accounted for the stability of the bacteriophages observed over the wide range of temperatures and pH values. A complete genomic and taxonomical characterization for all bacteriophages used in this study would be necessary to confirm this hypothesis.

When evaluated for their host specificities, it was determined that the bacteriophages were not only able to delay the growth of planktonic cells, but were also able to statistically reduce the relative abundance of *Pseudomonas*-related taxa within the biofilm (Table 3). Specifically, OTU analysis showed that *P. aeruginosa* was detrimentally impacted by the bacteriophages (Table S2). However, it was also observed that other taxa such as unclassified *Alphaproteobacteria* and *Rhodobacteraceae* decreased in their relative abundance. Bacteriophages used in this study showed no infectivity against non-*P. aeruginosa* bacterial hosts. This means that the effect on other bacteria within the biofilm matrix was likely due to an indirect action rather than a direct virus infection. Indeed, these two taxa are within the most abundant *Proteobacteria* phylum, and the reduction may be due to the loss of bacterial cells upon bacteriophage-induced biofilm disruption. *Pseudoalteromonas* was also detrimentally impacted by bacteriophage application. This genus has been reported to play an important role in marine biofilm by contributing to the EPS production (Dheilly et al., 2010; Liu et al., 2016) and its decrease in relative abundance reiterate the effect of bacteriophages on biofilm matrix. Finally, the decrease in relative abundance of Gram-positive *Planococcaceae* could be due to a higher susceptibility to phage lysins since in the absence of outer membrane, those proteins can make direct contact with cell wall carbohydrates and peptidoglycans even if *Planococcaceae* is not the target host (Fischetti, 2005).

Developing resistance to bacteriophage remains to be of a concern. This was exemplified when bacteria eventually overcame the bacteriophage to grow albeit at a lower specific growth rate compared to the same host grown in absence of bacteriophage (Table 2). Similar trend was also observed in a separate study for *P. aeruginosa* infected with *Podoviridae* phages (Alves et al., 2016). The bacterial growth after a prolonged lag phase indicates the evolution of resistance to bacteriophage infection after a certain period of bacteria-virus interaction. Small gene mutation or change/loss of receptor proteins among a subpopulation of cells can result in the loss of bacteriophage infectivity (Ly-Chatain, 2014). Bacterial resistance to bacteriophage infection can also occur by the production of competitive inhibitors that bind to phage receptor or by preventing phage DNA entry (Labrie et al., 2010). It was observed that *P. aeruginosa* phage resistance can affect also the expression of virulence factors (Hosseinidoust et al., 2013a,b) but incurs a fitness cost for the bacteria in terms of specific growth rate and metabolic activity (Hall et al., 2012). This can potentially account for the lower specific bacterial growth rates observed in presence of bacteriophages (Table 2). Besides gaining resistance, bacterial regrowth could possibly be related to the establishment of phage lysogeny which leads to the presence of prophages in the bacterial cells instead of cell lysis, hence allowing bacterial regrowth. Further studies would have to be carried out to determine the cause of this bacterial regrowth observed in presence of bacteriophages.

Subsequently, when bacteriophages were applied to the *P. aeruginosa*-enriched biofilm attached on UF membranes, it was observed that the starting TMP at each initial point of cleaning cycle was higher than the previous cycle (Figures S4, S5). This suggests a slight loss in bacteriophage efficacy with each application. Since the slopes of TMP increase after each cleaning cycle were not significantly different, the increase of initial TMP at each cleaning cycle was most likely not due to changes in metabolic rates or infectivity rates. Instead, this can potentially be explained by an increased production of EPS that would provide a physical barrier between bacteriophages and their receptors (Labrie et al., 2010). Earlier studies have reported a higher reduction in TMP decline compared to this study (i.e., ca. 18% decrease). For instance, Goldman and colleagues observed a 47% reduction in the UF permeability drop after phage application compared with the control (Goldman et al., 2009). However, bacteriophages were introduced in the feed solution and not directly on the formed biofilm, and the higher reduction may be due to a combined effect on both planktonic and biofilm-associated bacteria. In a full-scale SWRO, direct application of bacteriophage to a large volume of seawater would be impractical and costly as this means a large dose of bacteriophage would be required to maintain infectivity at an optimal MOI. In another study, Bhattacharjee et al. observed a 53 and 78% flux recovery after 1 and 2 days of phage application, respectively (Bhattacharjee et al., 2015). However, hollow-fiber membranes were conditioned with nutrient rich medium instead of environmental waters, and both bacteriophages and host may be more metabolically active to facilitate interactions.

Isolation of new bacteriophages or application of phages cocktail can be adopted to mitigate problems associated with phage resistance and lysogeny. However, isolating new bacteriophages can be challenging and time-consuming for long-term operations. Alternatively, this study demonstrated the feasibility of combining bacteriophages application with the common membrane cleaning procedure. Approximately 49% TMP recovery and 1.1 log biofilm removal was observed when UF membrane was treated with bacteriophages and citric acid, regardless of the order of application (Figure 3). This confirms that bacteriophages are not affected by the acidic pH and that they can be easily integrated with the common membrane treatment procedure to enhance the cleaning efficacy. Citric acid chelates inorganic minerals and disrupts the stability of biofilm matrix attached on the pretreatment UF membranes (Lee et al., 2001; Porcelli and Judd, 2010). Bacteriophages are able to further disrupt the biofilm structure, hence allowing for a better cleaning efficacy. Similarly, bacteriophages were applied successfully in combination with chlorine treatment against *P. aeruginosa* biofilm (Zhang and Hu, 2013). These findings suggest that bacteriophage can be applied synergistically with existing biocides or cleaning strategies to effectively mitigate biofouling associated with *P. aeruginosa*. Alternatively, other bacteriophages specific against other host targets, for example *Pseudoalteromonas* which is typically present in high abundance on a marine biofilm layer, can also be isolated and verified for their lytic efficiency against this new host based on the procedure described in this study. Finally, it could be interesting to analyze the effect of bacteriophages treatment in preventing biofilm formation rather than removing an established biofilm structure. In this context, the lower cell density could represent a limitation for bacteriophages activity and infectivity. A cocktail of bacteriophages from different taxonomical families targeting different bacterial host could also represent a better approach to maximize the impact of the infection and to further mitigate seawater membrane biofouling in the future for bigger scale operating systems.

## Conclusion

This study demonstrated the ability of bacteriophages to infect planktonic *P. aeruginosa* and to reduce biofilm formation. Infectivity at different environmental conditions was systematically evaluated and bacteriophages showed great versatility to infect *P. aeruginosa* over a wide temperature and pH range. Although the bacteriophages were able to effectively reduce the relative abundance of *Pseudomonas*-related taxa, other taxa including *Pseudoalteromonas* and predominant *Proteobacteria* within a marine biofilm were also affected, suggesting that the bacteriophages lyse *P. aeruginosa* and disrupt the biofilm matrix. Finally, bacteriophages were demonstrated to be feasible for reducing *P. aeruginosa* biofouling on a lab-scale UF membrane. Specifically, the best reduction in transmembrane pressure was obtained when bacteriophage treatment was combined with citric acid cleaning. Collectively, the findings demonstrate that bacteriophages can be used as a biocidal agent to mitigate undesirable *P. aeruginosa*-associated problems in seawater applications.

## Author contributions

GS and P-YH: Designed the experiment; GS and SY: Performed the experiments and analyzed the data; GS, P-YH, and AK: Wrote and revised the manuscript.

### Conflict of interest statement

The authors declare that the research was conducted in the absence of any commercial or financial relationships that could be construed as a potential conflict of interest.

## Supplementary material

Table S1 Similarity percentages (SIMPER) analysis of the average composition of the microbial communities in phage infected and non-infected biofilms.

Table S2 OTUs affected by bacteriophage infection are listed together with the closest match identified by the BLASTN algorithm.

Figure S1 Phage forming units for each bacteriophage propagated with the double layer plate method. Bars indicate standard deviation.

Figure S2 Cross-flow ultrafiltration membrane setup.

Figure S3 Values of TMP/TMP_0_ increment slopes, averaged from the three cycles of UF membrane cleaning. a, b represent homogenous subgroups by two-tailed *t*-test.

Figure S4 Values of TMP/TMP_0_ increment slopes for each cycle of membrane cleaning.

Figure S5 Run 2 of the ultrafiltration membrane experiment. **(A)** Transmembrane pressure (TMP) profiles of ultrafiltration membranes. TMP was normalized against the initial TMP (TMP_0_) for each membrane. Different treatments were applied, namely CA, Citric acid; PH, Phage; CA+PH, Citric acid followed by phage; PH+CA, Phage followed by citric acid; C, no treatment. Dashed red lines indicate the point at which treatment was applied over three different cycles. **(B)** Cell numbers from plate counts in terms of CFU/mL, **(C)** and from flow cytometry in terms of cells number/mL, were measured on three 2 × 2 cm pieces for each membrane at the end of the experiment. Bars indicate standard deviation among the three biological replicates. Letters indicate statistical difference compared with the control (a: *p* < 0.0006, b: *p* < 0.007 and c: *p* < 0.05).

Figure S6 Pulsed field gel electrophoresis images of P1, P5, and P7 genomes. λ and a indicate *lambda* and 1 kbp ladder respectively used as markers.

Figure S7 Transmission electron microscopy images of the isolated bacteriophage P1.

Figure S8 Transmission electron microscopy images of the isolated bacteriophage P5.

Figure S9 Transmission electron microscopy images of the isolated bacteriophage P7 in aggregate.

Figure S10 Transmission electron microscopy images of the isolated bacteriophage P7.

## References

[B1] Ackermann H. W. (2006). Classification of bacteriophages, in The Bacteriophages, ed Calendar R. (New York, NY: Oxford University Press), 8–16.

[B2] Adams M. H. (1959). Bacteriophages. New York, NY: Interscience Publishers Inc.

[B3] Alemayehu D. Casey P. G. McAuliffe O. Guinane C. M. Martin J. G. Shanahan F.. (2012). Bacteriophages φMR299-2 and φNH-4 can eliminate *Pseudomonas aeruginosa* in the murine lung and on cystic fibrosis lung airway cells. MBio 3, e00029–e00012. 10.1128/mBio.00029-12 22396480 PMC3302570

[B4] Al-Jassim N. Ansari M. I. Harb M. Hong P.-Y. (2015). Removal of bacterial contaminants and antibiotic resistance genes by conventional wastewater treatment processes in Saudi Arabia: is the treated wastewater safe to reuse for agricultural irrigation? Water Res. 73, 277–290. 10.1016/j.watres.2015.01.036 25687420

[B5] Alves D. R. Perez-Esteban P. Kot W. Bean J. Arnot T. Hansen L. H.. (2016). A novel bacteriophage cocktail reduces and disperses *Pseudomonas aeruginosa* biofilms under static and flow conditions. Microb. Biotechnol. 9, 61–74. 10.1111/1751-7915.12316 26347362 PMC4720417

[B6] Bhattacharjee A. S. Choi J. Motlagh A. M. Mukherji S. T. Goel R. (2015). Bacteriophage therapy for membrane biofouling in membrane bioreactors and antibiotic-resistant bacterial biofilms. Biotechnol. Bioeng. 112, 1644–1654. 10.1002/bit.25574 25728819

[B7] Chibani-Chennoufi S. Bruttin A. Dillmann M.-L. Brüssow H. (2004). Phage-host interaction: an ecological perspective. J. Bacteriol. 186, 3677–3686. 10.1128/JB.186.12.3677-3686.2004 15175280 PMC419959

[B8] Clarke K. G. R. (2015). PRIMER Version 7: User Manual/Tutorial. Plymouth: PRIMER-E.

[B9] Dheilly A. Soum-Soutéra E. Klein G. L. Bazire A. Compère C. Haras D.. (2010). Antibiofilm activity of the marine bacterium *Pseudoalteromonas* sp. strain 3J6. Appl. Environ. Microbiol. 76, 3452–3461. 10.1128/AEM.02632-09 20363799 PMC2876442

[B10] Echols H. (1972). Developmental pathways for the temperate phage: lysis vs lysogeny. Annu. Rev. Genet. 6, 157–190. 10.1146/annurev.ge.06.120172.001105 4604314

[B11] Edgar R. C. Haas B. J. Clemente J. C. Quince C. Knight R. (2011). UCHIME improves sensitivity and speed of chimera detection. Bioinformatics 27, 2194–2200. 10.1093/bioinformatics/btr381 21700674 PMC3150044

[B12] Fischetti V. A. (2005). Bacteriophage lytic enzymes: novel anti-infectives. Trends Microbiol. 13, 491–496. 10.1016/j.tim.2005.08.007 16125935

[B13] Fritzmann C. Löwenberg J. Wintgens T. Melin T. (2007). State-of-the-art of reverse osmosis desalination. Desalination 216, 1–76. 10.1016/j.desal.2006.12.009

[B14] Fu W. Forster T. Mayer O. Curtin J. J. Lehman S. M. Donlan R. M. (2010). Bacteriophage cocktail for the prevention of biofilm formation by *Pseudomonas aeruginosa* on catheters in an *in vitro* model system. Antimicrob. Agents Chemother. 54, 397–404. 10.1128/AAC.00669-09 19822702 PMC2798481

[B15] Gao W. Liang H. Ma J. Han M. Chen Z.-L. Han Z.-S. (2011). Membrane fouling control in ultrafiltration technology for drinking water production: a review. Desalination 272, 1–8. 10.1016/j.desal.2011.01.051

[B16] Goldman G. Starosvetsky J. Armon R. (2009). Inhibition of biofilm formation on UF membrane by use of specific bacteriophages. J. Memb. Sci. 342, 145–152. 10.1016/j.memsci.2009.06.036

[B17] Goosen M. Sablani S. Al-Hinai H. Al-Obeidani S. Al-Belushi R. Jackson D. (2005). Fouling of reverse osmosis and ultrafiltration membranes: a critical review. Sep. Sci. Technol. 39, 2261–2297. 10.1081/SS-120039343

[B18] Greenlee L. F. Lawler D. F. Freeman B. D. Marrot B. Moulin P. (2009). Reverse osmosis desalination: water sources, technology, and today's challenges. Water Res. 43, 2317–2348. 10.1016/j.watres.2009.03.010 19371922

[B19] Hall A. R. De Vos D. Friman V.-P. Pirnay J.-P. Buckling A. (2012). Effects of sequential and simultaneous applications of bacteriophages on populations of *Pseudomonas aeruginosa in vitro* and in wax moth larvae. Appl. Environ. Microbiol. 78, 5646–5652. 10.1128/AEM.00757-12 22660719 PMC3406105

[B20] Harper D. R. Parracho H. M. Walker J. Sharp R. Hughes G. Werthén M. (2014). Bacteriophages and biofilms. Antibiotics 3, 270–284. 10.3390/antibiotics3030270

[B21] Hong P.-Y. Moosa N. Mink J. (2016). Dynamics of microbial communities in an integrated ultrafiltration–reverse osmosis desalination pilot plant located at the Arabian Gulf. Desalination Water Treat. 57, 16310–16323. 10.1080/19443994.2015.1083483

[B22] Hosseinidoust Z. Tufenkji N. Van De Ven T. G. (2013a). Predation in homogeneous and heterogeneous phage environments affects virulence determinants of *Pseudomonas aeruginosa*. Appl. Environ. Microbiol. 79, 2862–2871. 10.1128/AEM.03817-12 23435883 PMC3623153

[B23] Hosseinidoust Z. Van De Ven T. G. Tufenkji N. (2013b). Evolution of *Pseudomonas aeruginosa* virulence as a result of phage predation. Appl. Environ. Microbiol. 79, 6110–6116. 10.1128/AEM.01421-13 23892756 PMC3811365

[B24] Jonczyk E. Kłak M. Miedzybrodzki R. Górski A. (2011). The influence of external factors on bacteriophages. Folia Microbiol. 56, 191–200. 10.1007/s12223-011-0039-8 21625877 PMC3131515

[B25] Khan M. T. Hong P.-Y. Nada N. Croue J. P. (2015). Does chlorination of seawater reverse osmosis membranes control biofouling? Water Res. 78, 84–97. 10.1016/j.watres.2015.03.029 25917390

[B26] Kot W. Vogensen F. K. Sørensen S. J. Hansen L. H. (2014). DPS–a rapid method for genome sequencing of DNA-containing bacteriophages directly from a single plaque. J. Virol. Methods 196, 152–156. 10.1016/j.jviromet.2013.10.040 24239631

[B27] Kwiatek M. Mizak L. Parasion S. Gryko R. Olender A. Niemcewicz M. (2015). Characterization of five newly isolated bacteriophages active against *Pseudomonas aeruginosa* clinical strains. Folia Microbiol. 60, 7–14. 10.1007/s12223-014-0333-3 24993480

[B28] Labrie S. J. Samson J. E. Moineau S. (2010). Bacteriophage resistance mechanisms. Nat. Rev. Microbiol. 8, 317–327. 10.1038/nrmicro2315 20348932

[B29] Langlet J. Gaboriaud F. Gantzer C. (2007). Effects of pH on plaque forming unit counts and aggregation of MS2 bacteriophage. J. Appl. Microbiol. 103, 1632–1638. 10.1111/j.1365-2672.2007.03396.x 17953574

[B30] Lee H. Amy G. Cho J. Yoon Y. Moon S.-H. Kim I. S. (2001). Cleaning strategies for flux recovery of an ultrafiltration membrane fouled by natural organic matter. Water Res. 35, 3301–3308. 10.1016/S0043-1354(01)00063-X 11547850

[B31] Le Roux J. Nada N. Khan M. T. Croué J.-P. (2015). Tracing disinfection byproducts in full-scale desalination plants. Desalination 359, 141–148. 10.1016/j.desal.2014.12.035

[B32] Liu A. Mi Z.-H. Zheng X.-Y. Yu Y. Su H.-N. Chen X.-L.. (2016). Exopolysaccharides play a role in the swarming of the benthic bacterium *Pseudoalteromonas* sp. SM9913. Front. Microbiol. 7:473. 10.3389/fmicb.2016.00473 27092127 PMC4820436

[B33] Ly-Chatain M. H. (2014). The factors affecting effectiveness of treatment in phages therapy. Front. Microbiol. 5:51. 10.3389/fmicb.2014.00051 24600439 PMC3927074

[B34] Matin A. Khan Z. Zaidi S. Boyce M. (2011). Biofouling in reverse osmosis membranes for seawater desalination: phenomena and prevention. Desalination 281, 1–16. 10.1016/j.desal.2011.06.063

[B35] McVay C. S. Velásquez M. Fralick J. A. (2007). Phage therapy of *Pseudomonas aeruginosa* infection in a mouse burn wound model. Antimicrob. Agents Chemother. 51, 1934–1938. 10.1128/AAC.01028-06 17387151 PMC1891379

[B36] Nap R. J. BoŽič A. L. Szleifer I. Podgornik R. (2014). The role of solution conditions in the bacteriophage PP7 capsid charge regulation. Biophys. J. 107, 1970–1979. 10.1016/j.bpj.2014.08.032 25418178 PMC4213675

[B37] Oh H.-S. Tan C. H. Low J. H. Rzechowicz M. Siddiqui M. F. Winters H.. (2017). Quorum quenching bacteria can be used to inhibit the biofouling of reverse osmosis membranes. Water Res. 112, 29–37. 10.1016/j.watres.2017.01.028 28129553

[B38] Olszak T. Zarnowiec P. Kaca W. Danis-Wlodarczyk K. Augustyniak D. Drevinek P.. (2015). *In vitro* and *in vivo* antibacterial activity of environmental bacteriophages against *Pseudomonas aeruginosa* strains from cystic fibrosis patients. Appl. Microbiol. Biotechnol. 99, 6021–6033. 10.1007/s00253-015-6492-6 25758956 PMC4480334

[B39] Peñate B. García-Rodríguez L. (2012). Current trends and future prospects in the design of seawater reverse osmosis desalination technology. Desalination 284, 1–8. 10.1016/j.desal.2011.09.010

[B40] Pires D. P. Boas D. V. Sillankorva S. Azeredo J. (2015). Phage therapy: a step forward in the treatment of *Pseudomonas aeruginosa* infections. J. Virol. 89, 7449–7456. 10.1128/JVI.00385-15 25972556 PMC4505681

[B41] Porcelli N. Judd S. (2010). Chemical cleaning of potable water membranes: a review. Sep. Purif. Technol. 71, 137–143. 10.1016/j.seppur.2009.12.007 20031188

[B42] Sanawar H. Xiong Y. Alam A. Croue J.-P. Hong P.-Y. (2017). Chlorination or monochloramination: balancing the regulated trihalomethane formation and microbial inactivation in marine aquaculture waters. Aquaculture 480, 94–102. 10.1016/j.aquaculture.2017.08.014

[B43] Scarascia G. Cheng H. Harb M. Hong P.-Y. (2017). Application of hierarchical oligonucleotide primer extension (HOPE) to assess relative abundances of ammonia-and nitrite-oxidizing bacteria. BMC Microbiol. 17:85. 10.1186/s12866-017-0998-2 28376730 PMC5381152

[B44] Sillankorva S. Oliveira R. Vieira M. J. Sutherland I. Azeredo J. (2004). Pseudomonas fluorescens infection by bacteriophage ΦS1: the influence of temperature, host growth phase and media. FEMS Microbiol. Lett. 241, 13–20. 10.1016/j.femsle.2004.06.058 15556704

[B45] Wang Q. Garrity G. M. Tiedje J. M. Cole J. R. (2007). Naive Bayesian classifier for rapid assignment of rRNA sequences into the new bacterial taxonomy. Appl. Environ. Microbiol. 73, 5261–5267. 10.1128/AEM.00062-07 17586664 PMC1950982

[B46] Young R. (2013). Phage lysis: do we have the hole story yet? Curr. Opin. Microbiol. 16, 790–797. 10.1016/j.mib.2013.08.008 24113139 PMC3848059

[B47] Young R. (2014). Phage lysis: three steps, three choices, one outcome. J. Microbiol. 52, 243–258. 10.1007/s12275-014-4087-z 24585055 PMC4012431

[B48] Zhang Y. Hunt H. K. Hu Z. (2013). Application of bacteriophages to selectively remove *Pseudomonas aeruginosa* in water and wastewater filtration systems. Water Res. 47, 4507–4518. 10.1016/j.watres.2013.05.014 23764600

[B49] Zhang Y. Hu Z. (2013). Combined treatment of *Pseudomonas aeruginosa* biofilms with bacteriophages and chlorine. Biotechnol. Bioeng. 110, 286–295. 10.1002/bit.24630 22886888

