# Supplementary material for: Bacteriophage Infectivity Against Pseudomonas aeruginosa in Saline Conditions
Source: Front Microbiol. 2018 May 2;9:875. doi: 10.3389/fmicb.2018.00875 (PMC5942161; doi:10.3389/fmicb.2018.00875)
Supplement: Table S1 — Similarity percentages (SIMPER) analysis of the average composition of the microbial communities in phage infected and non-infected biofilms. [file Table_1.DOCX]

**Table S1.** Similarity percentages (SIMPER) analysis of the average composition of the microbial communities in phage infected and non-infected biofilms. Only the top 10 bacterial populations impacted by bacteriophages application are shown. Groups P1, P5 and P7 denote the bacteriophages used as treatment. Group C denotes control. Grey shaded rows denote the *Pseudomonas*-related taxa.

|  | **Taxa** | **Average Abundance** | **Average Abundance** | **Average Dissimilarity** | **Contribution %** | **Cumulative%** |
| --- | --- | --- | --- | --- | --- | --- |
| **P1 & C average dissimilarity: 14.69%** |  | Group P1 | Group C |  |  |  |
|  | Unclassified *Pseudomonadaceae* | 0.41 | 1.59 | 1.2 | 8.14 | 8.14 |
|  | *Pseudomonas* | 0.4 | 1.42 | 1.04 | 7.08 | 15.22 |
|  | *Pseudoalteromonas* | 4.33 | 3.42 | 0.93 | 6.34 | 21.56 |
|  | Unclassified *Planococcaceae* | 1.66 | 0.76 | 0.93 | 6.3 | 27.86 |
|  | Unclassified *Alphaproteobacteria* | 1.12 | 1.7 | 0.59 | 4.05 | 31.91 |
|  | Unclassified *Rhodobacteraceae* | 0.79 | 1.26 | 0.48 | 3.28 | 35.19 |
|  | Unclassified *Proteobacteria* | 0.91 | 1.38 | 0.48 | 3.28 | 38.47 |
|  | Unclassified *Bacteria* | 0.95 | 1.39 | 0.45 | 3.05 | 41.52 |
|  | *Sneathiella* | 0.75 | 1.18 | 0.45 | 3.03 | 44.55 |
|  | *Thalassospira* | 1.24 | 0.85 | 0.4 | 2.71 | 47.26 |
| **P5 & C average dissimilarity: 17.42%** |  | Group P5 | Group C |  |  |  |
|  | Unclassified *Planococcaceae* | 1.98 | 0.76 | 1.27 | 7.28 | 7.28 |
|  | Unclassified *Pseudomonadaceae* | 0.38 | 1.59 | 1.25 | 7.17 | 14.45 |
|  | *Pseudomonas* | 0.36 | 1.42 | 1.1 | 6.29 | 20.74 |
|  | *Pseudoalteromonas* | 4.24 | 3.42 | 0.85 | 4.88 | 25.62 |
|  | Unclassified *Proteobacteria* | 0.79 | 1.38 | 0.61 | 3.5 | 29.12 |
|  | Unclassified *Alphaproteobacteria* | 1.12 | 1.7 | 0.6 | 3.46 | 32.58 |
|  | *Alcanivorax* | 0.71 | 1.24 | 0.55 | 3.18 | 35.75 |
|  | *Sneathiella* | 0.7 | 1.18 | 0.49 | 2.84 | 38.59 |
|  | Unclassified *Rhodobacteraceae* | 0.79 | 1.26 | 0.49 | 2.8 | 41.39 |
|  | *Terasakiella* | 0.8 | 1.27 | 0.49 | 2.79 | 44.17 |
| **P7 & C average dissimilarity: 13.78%** |  | Group P7 | Group C |  |  |  |
|  | Unclassified *Planococcaceae* | 2.23 | 0.76 | 1.49 | 10.8 | 10.8 |
|  | Unclassified *Pseudomonadaceae* | 0.42 | 1.59 | 1.18 | 8.57 | 19.36 |
|  | *Pseudomonas* | 0.4 | 1.42 | 1.03 | 7.5 | 26.86 |
|  | *Pseudoalteromonas* | 4.14 | 3.42 | 0.73 | 5.28 | 32.14 |
|  | *Terasakiella* | 0.69 | 1.27 | 0.59 | 4.24 | 36.38 |
|  | Unclassified *Alphaproteobacteria* | 1.3 | 1.7 | 0.41 | 2.97 | 39.35 |
|  | Unclassified *Proteobacteria* | 1.07 | 1.38 | 0.31 | 2.27 | 41.62 |
|  | *Aestuariibacter* | 0.21 | 0.52 | 0.31 | 2.27 | 43.9 |
|  | *Sneathiella* | 0.89 | 1.18 | 0.3 | 2.15 | 46.05 |
|  | Unclassified *Rhodobacteraceae* | 0.97 | 1.26 | 0.29 | 2.1 | 48.15 |

**Table S2.** OTUs affected by bacteriophage infection are listed together with the closest match identified by the BLASTN algorithm. The average relative abundance is calculated among the three biological replicates. Group P1, P5 and P7 denote the bacteriophage used as treatment. Group C denotes control. Grey shaded rows denote the *Pseudomonas*-related taxa.

| **OTU** | **Average Abundance** | **Average Abundance** | **Bacteria** | **Score** | **Query cover** | **E-value** | **Identity** |
| --- | --- | --- | --- | --- | --- | --- | --- |
|  | Group P1 | Group C |  |  |  |  |  |
|  |  |  |  |  |  |  |  |
| OTU968 | 0.35 | 0.81 | *Pseudomonas aeruginosa* | 499 | 97% | 3.00E-141 | 98% |
| OTU1725 | 0.38 | 2.14 | *Pseudomonas aeruginosa* | 534 | 100% | 8.00E-152 | 99% |
| OTU951 | 4.46 | 3.95 | *Pseudoalteromonas shioyasakiensis* | 503 | 100% | 2.00E-142 | 98% |
| OTU1718 | 3.32 | 2.13 | *Pseudoalteromonas shioyasakiensis* | 540 | 100% | 2.00E-153 | 99% |
| OTU1832 | 0.72 | 0.41 | *Thalassospira tiepidiphila* | 527 | 100% | 1.00E-149 | 100% |
|  | Group P5 | Group C |  |  |  |  |  |
| OTU968 | 0.35 | 0.81 | *Pseudomonas aeruginosa* | 499 | 97% | 3.00E-141 | 98% |
| OTU1725 | 0.33 | 2.14 | *Pseudomonas aeruginosa* | 534 | 100% | 8.00E-152 | 99% |
| OTU951 | 4.55 | 3.95 | *Pseudoalteromonas shioyasakiensis* | 503 | 100% | 2.00E-142 | 98% |
| OTU1718 | 2.89 | 2.13 | *Pseudoalteromonas shioyasakiensis* | 540 | 100% | 2.00E-153 | 99% |
| OTU972 | 0.27 | 0.55 | *Alcanivorax xenomutans* | 529 | 100% | 4.00E-150 | 99% |
|  | Group P7 | Group C |  |  |  |  |  |
| OTU968 | 0.31 | 0.81 | *Pseudomonas aeruginosa* | 499 | 97% | 3.00E-141 | 98% |
| OTU1725 | 0.48 | 2.14 | *Pseudomonas aeruginosa* | 534 | 100% | 8.00E-152 | 99% |
| OTU951 | 4.14 | 3.95 | *Pseudoalteromonas shioyasakiensis* | 503 | 100% | 2.00E-142 | 98% |
| OTU1718 | 3.5 | 2.13 | *Pseudoalteromonas shioyasakiensis* | 540 | 100% | 2.00E-153 | 99% |
| OTU1744 | 0.13 | 0.4 | *Aestuatiibacter aggregatus* | 497 | 100% | 1.00E-140 | 98% |
|  |  |  |  |  |  |  |  |
