# Supplementary material for: Bacteriophage Infectivity Against Pseudomonas aeruginosa in Saline Conditions
Source: Front Microbiol. 2018 May 2;9:875. doi: 10.3389/fmicb.2018.00875 (PMC5942161; doi:10.3389/fmicb.2018.00875)
Supplement: Figure S1 — Phage forming units for each bacteriophage propagated with the double layer plate method. Bars indicate standard deviation. [file Image_1.PDF]

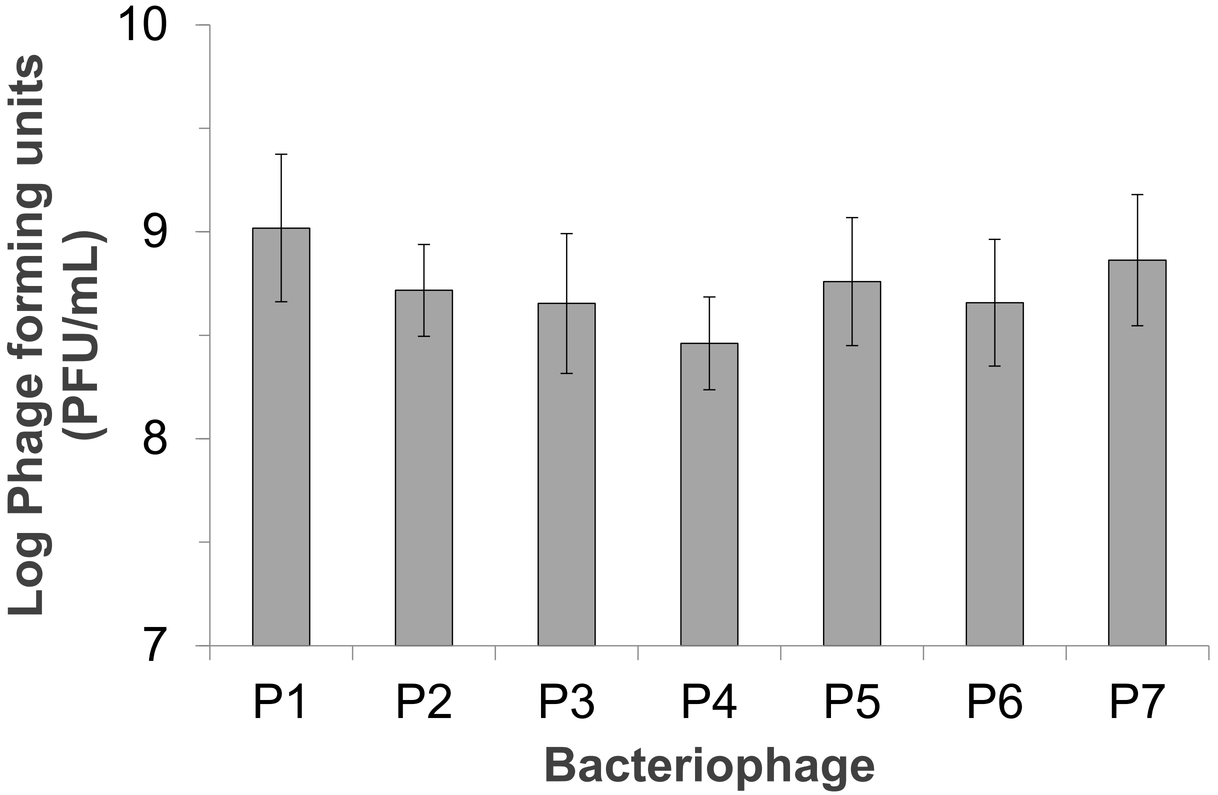

Figure S1. Phage forming units for each bacteriophage propagated with the double layer plate method. Bars indicate standard deviation.

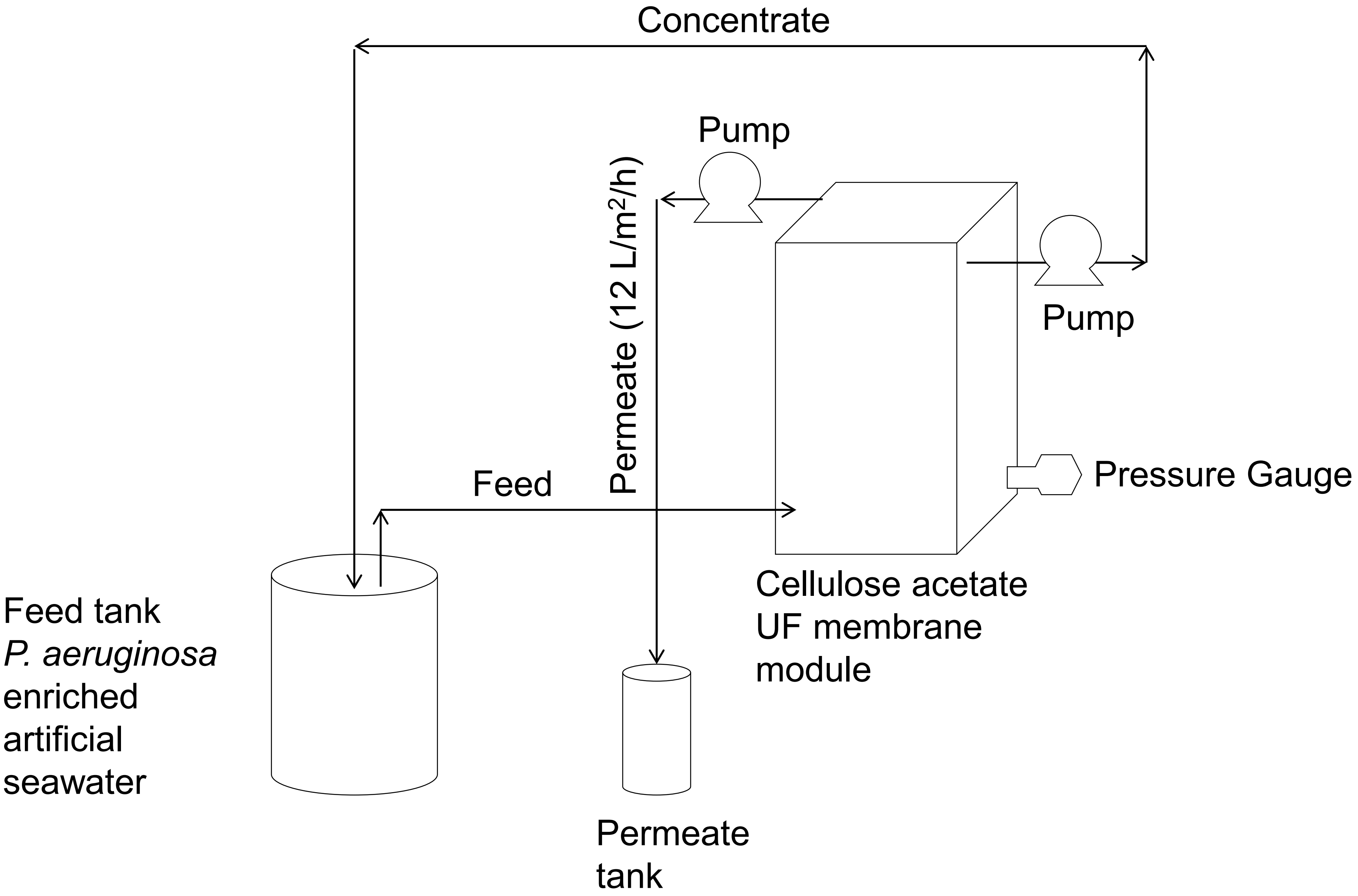

Figure S2. Cross-flow ultrafiltration membrane setup.

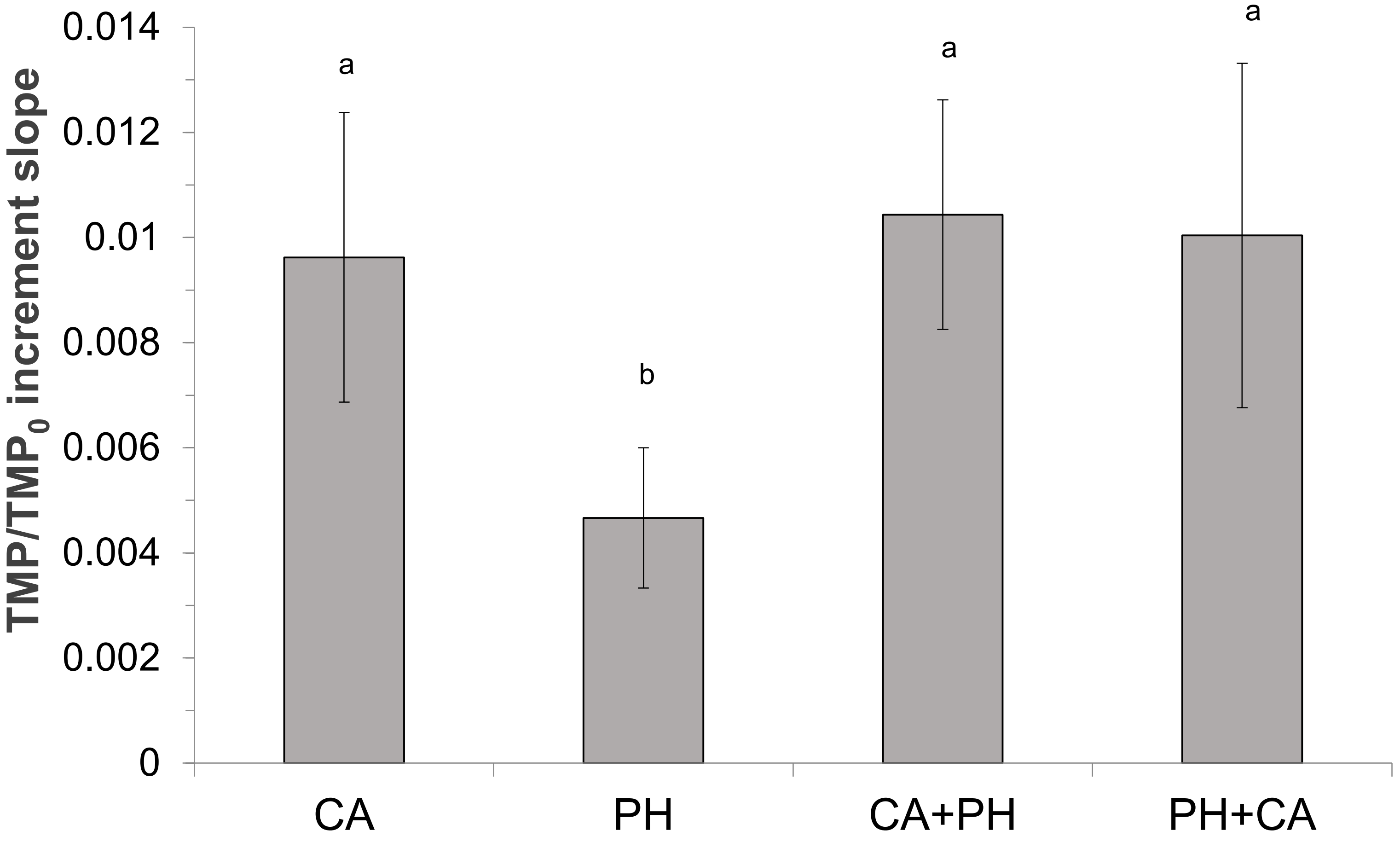

Figure S3. Values of TMP/TMP_0 increment slopes, averaged from the three cycles of UF membrane cleaning.
a, b represent homogenous subgroups by two-tailed t-test.

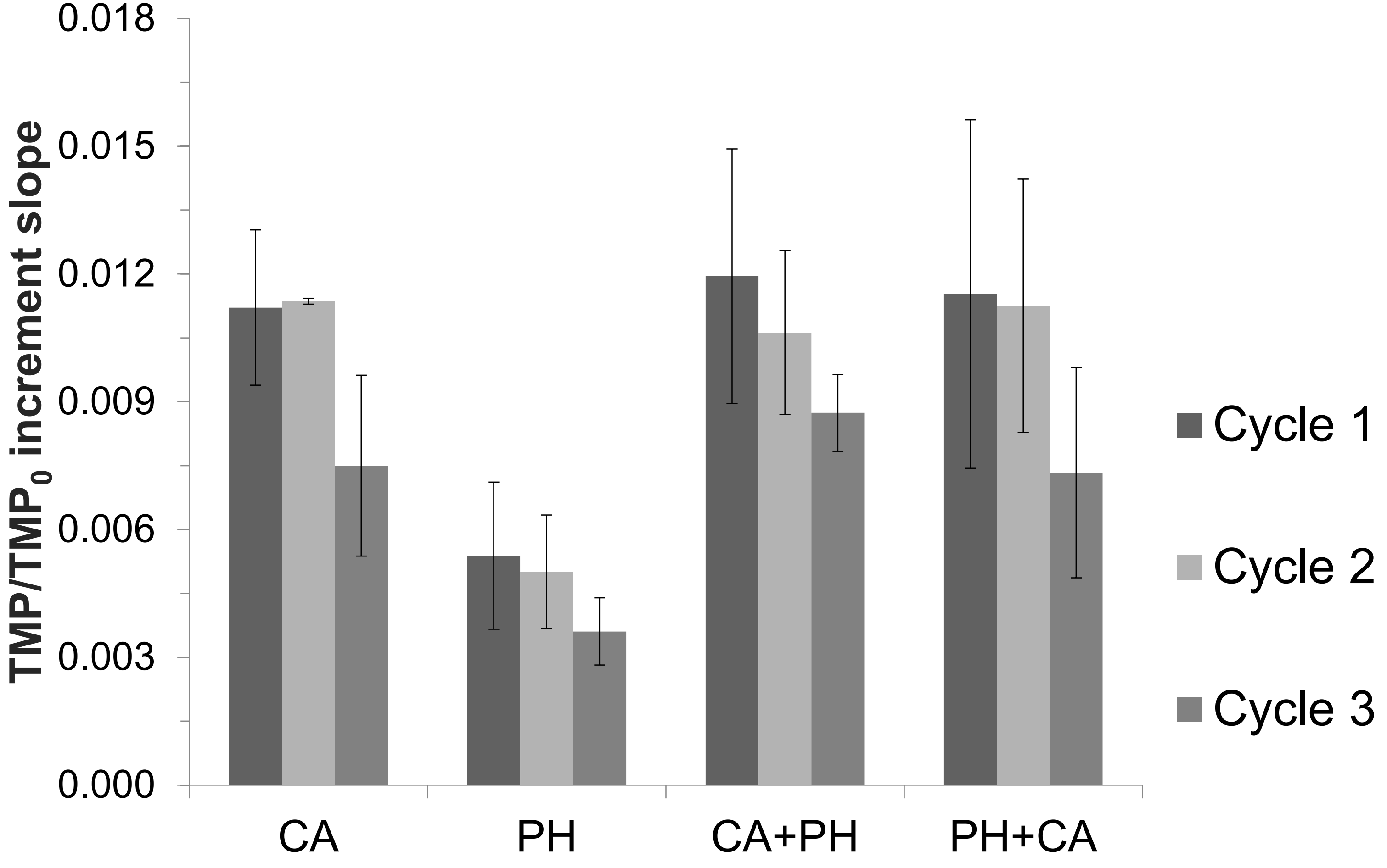

Figure S4. Values of TMP/TMP_0 increment slopes for each cycle of membrane cleaning.

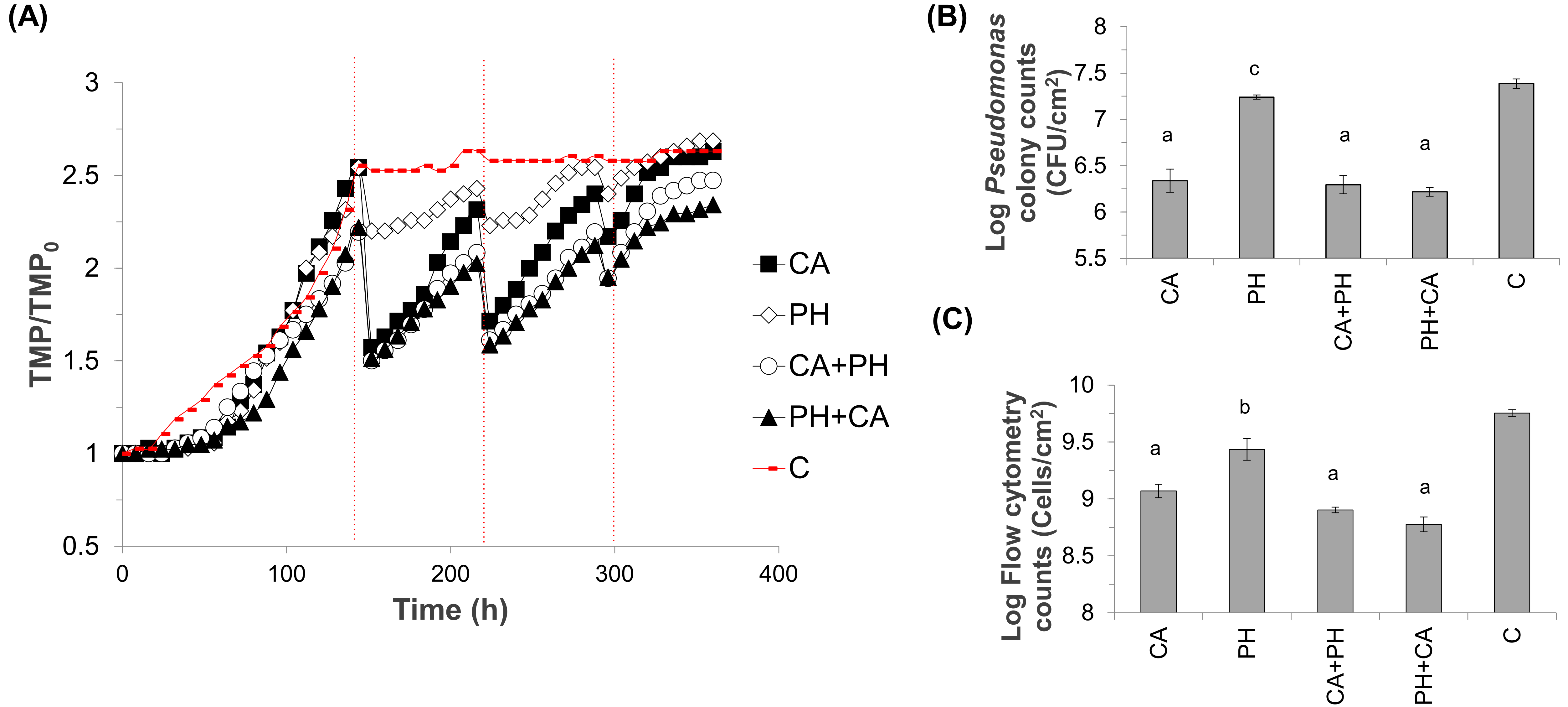

Figure S5. Run 2 of the ultrafiltration membrane experiment. **(A)** Transmembrane pressure (TMP) profiles of ultrafiltration membranes. TMP was normalized against the initial TMP (TMP_0) for each membrane. Different treatments were applied, namely CA = Citric acid; PH = Phage; CA+PH = Citric acid followed by phage; PH+CA = Phage followed by citric acid; C = no treatment. Dashed red lines indicate the point at which treatment was applied over three different cycles. **(B)** Cell numbers from plate counts in terms of CFU/mL, **(C)** and from flow cytometry in terms of cells number/mL, were measured on three 2x2 cm pieces for each membrane at the end of the experiment. Bars indicate standard deviation among the three biological replicates. Letters indicate statistical difference compared with the control (a: $p < 0.0006$, b: $p < 0.007$ and c: $p < 0.05$).

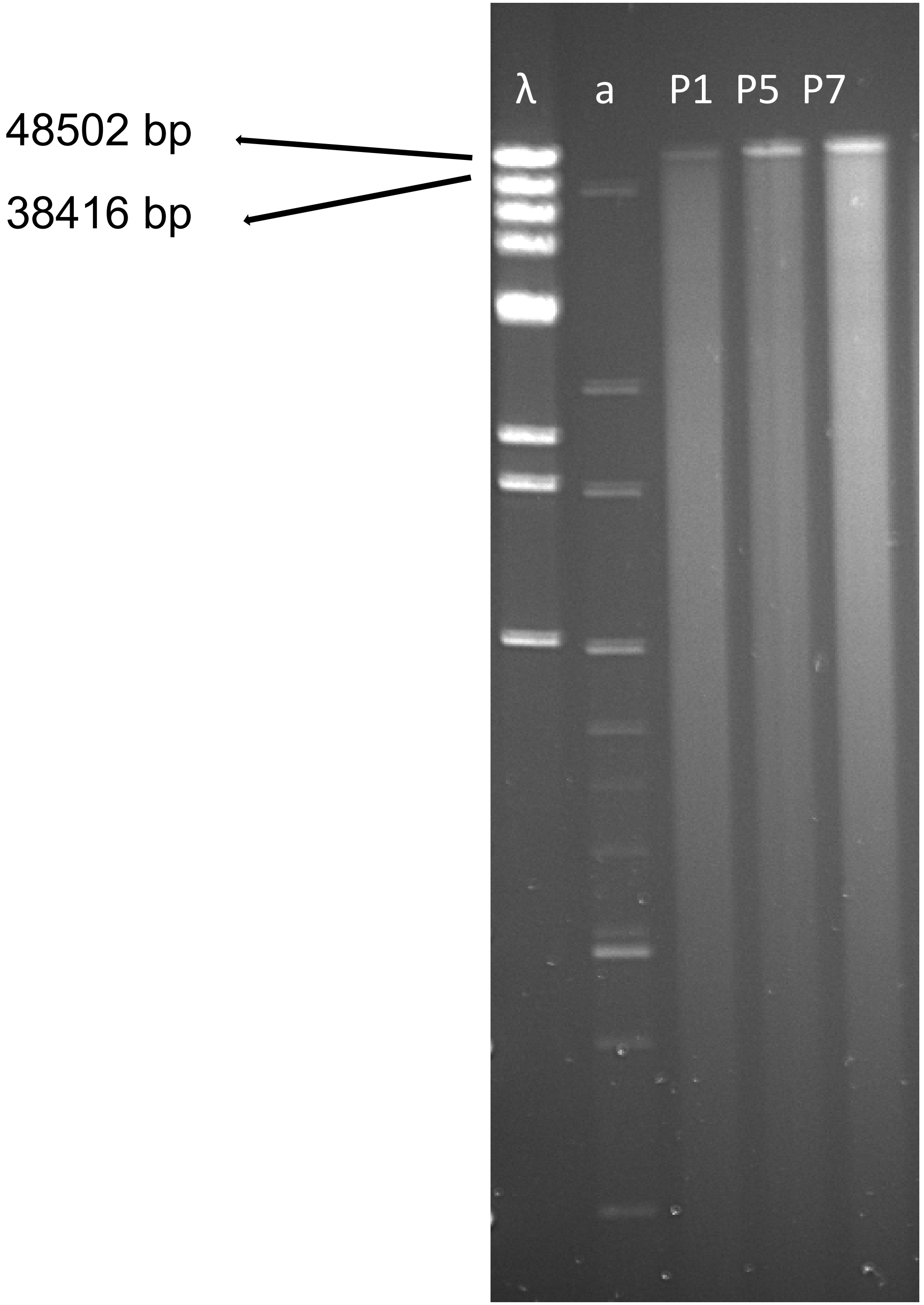

Figure S6. Pulsed field gel electrophoresis images of P1, P5 and P7 genomes. **λ** and a indicate *lambda* and 1 kbp ladder respectively used as markers.
