# Supplementary figures and images for: Bacteriophage Infectivity Against Pseudomonas aeruginosa in Saline Conditions
Source: Front Microbiol. 2018 May 2;9:875. doi: 10.3389/fmicb.2018.00875 (PMC5942161; doi:10.3389/fmicb.2018.00875)

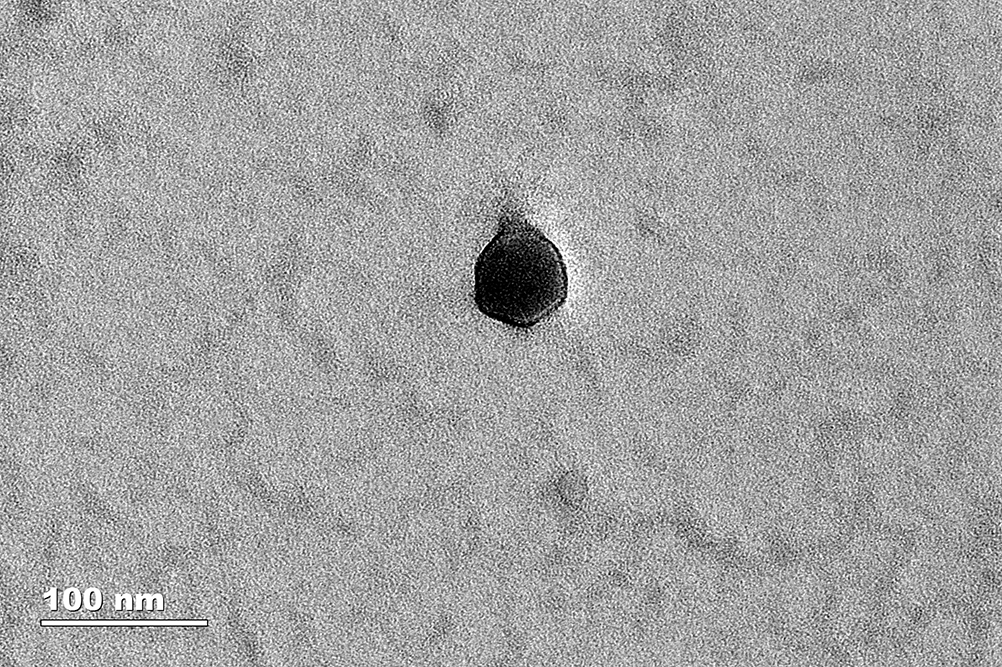

Supplement: Figure S7 — Transmission electron microscopy images of the isolated bacteriophage P1. [file Image_7.TIF]

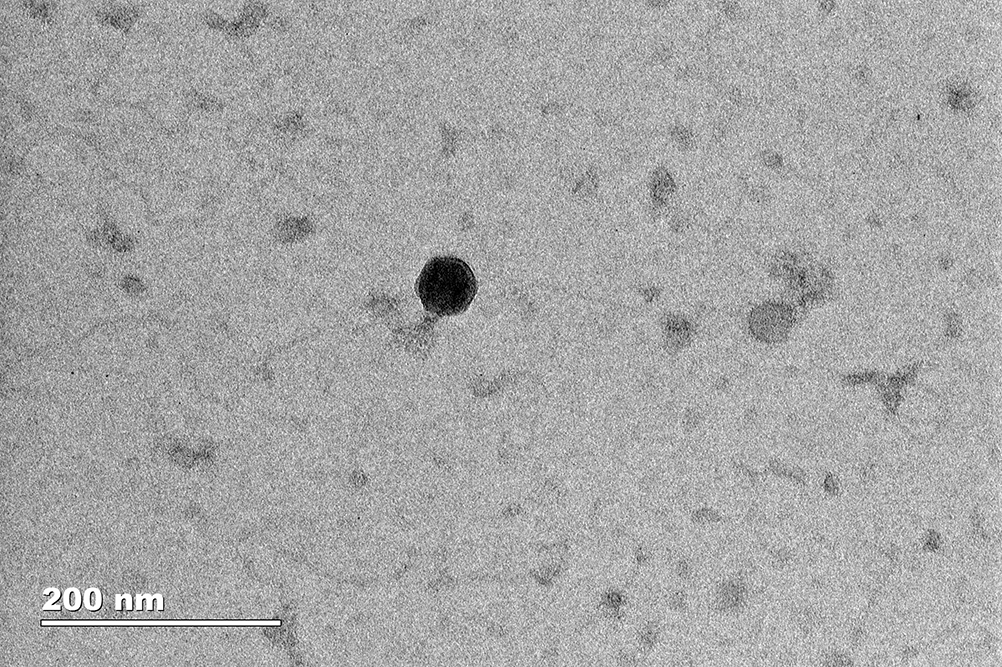

Supplement: Figure S8 — Transmission electron microscopy images of the isolated bacteriophage P5. [file Image_8.TIF]

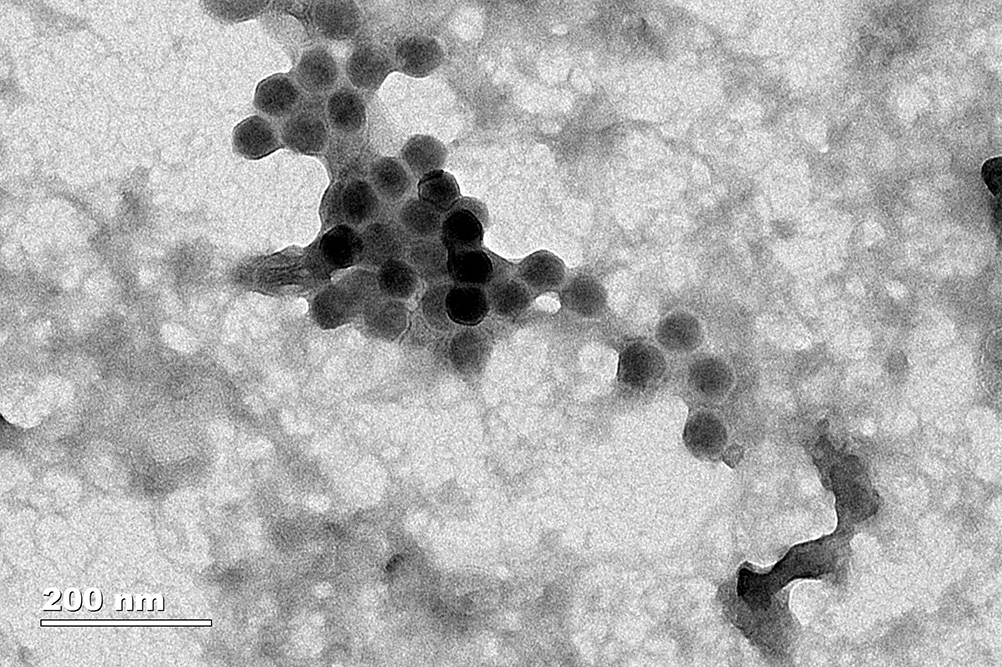

Supplement: Figure S9 — Transmission electron microscopy images of the isolated bacteriophage P7 in aggregate. [file Image_9.TIF]

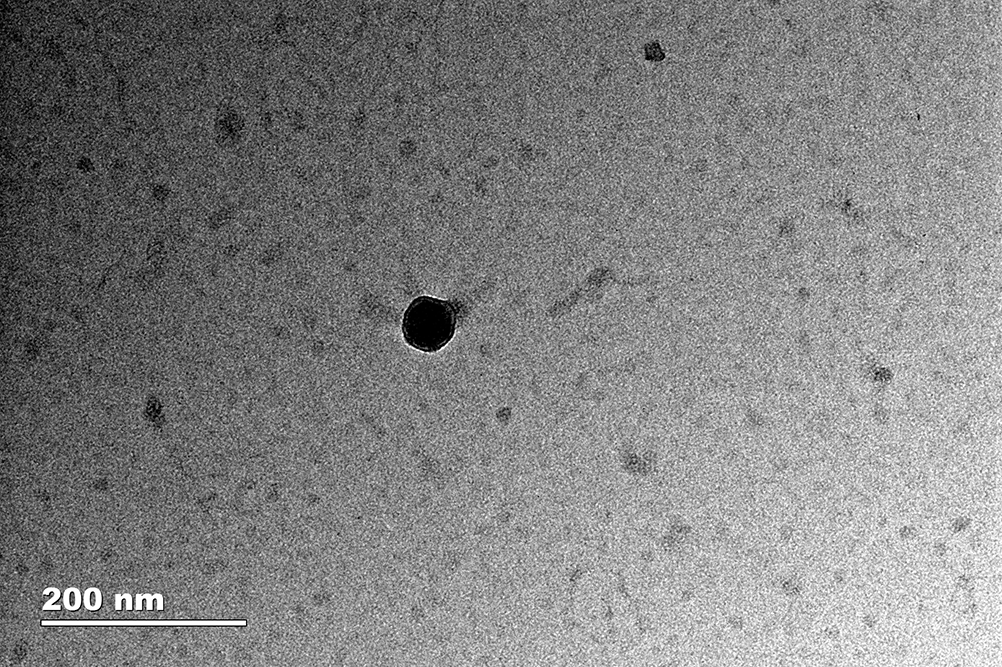

Supplement: Figure S10 — Transmission electron microscopy images of the isolated bacteriophage P7. [file Image_10.TIF]
